# *Lactiplantibacillusplantarum* HM-P2 influences gestational gut microbiome and microbial metabolism

**DOI:** 10.3389/fnut.2024.1489359

**Published:** 2024-12-20

**Authors:** Bin Liu, Zhenzhen Zhang, Junying Zhao, Xianping Li, Yaru Wang, Lu Liu, Weicang Qiao, Lijun Chen

**Affiliations:** ^1^National Engineering Research Center of Dairy Health for Maternal and Child, Beijing Sanyuan Foods Co. Ltd., Beijing, China; ^2^Beijing Engineering Research Center of Dairy, Beijing Technical Innovation Center of Human Milk Research, Beijing Sanyuan Foods Co. Ltd., Beijing, China

**Keywords:** probiotics, *Lactiplantibacillus plantarum* HM-P2, humanized germ-free mice, fecal microbiome transplantation, microbial metabolites

## Abstract

**Introduction:**

Human milk-derived probiotics are beneficial bacteria that provide gestational health benefits, for both pregnant women and their offspring. The study aims to investigate whether the administration of human milk-derived probiotic *L. plantarum* HM-P2 could effectively influence gestational health.

**Methods:**

The gestational humanized microbiome model was built by fecal microbiome transplant from gestational women into germ-free (GF) mice.

**Results:**

HM-P2 was successfully planted and increased the top crypt depth of the colon, and microbes such as *L. reuteri,Anaerofilum sp. An201,* and Gemmiger were up-regulated in the HM-P2 group throughout gestation. HM-P2 significantly promoted the contents of intestinal caproic acid, bile acids, and tryptophan catabolites such as serotonin. Gut microbes were associated with these bile acids and tryptophans.

**Discussion:**

HM-P2 could modulate the microbial community and microbial metabolites in gestational humanized GF mice. This probiotic strain could be a potential gestational dietary supplement with health benefits.

## Introduction

The use of probiotics during pregnancy has gained significant attention due to their potential health benefits. This can provide benefits to children by reducing instances of gastrointestinal disorders (1). *Lactiplantibacillus plantarum* is one of the most popular probiotics, with a versatile species/strain with beneficial qualities that are typically present in a wide variety of fermented food products (2). Furthermore, *L. plantarum* is generally considered safe and is commonly used in industrial fermentation and raw food processing.

*Lactiplantibacillus plantarum* can colonize the mice’s digestive tracts and affect their growth and immune responses (3). It can also reduce ulcerative colitis by improving gut inflammation and restoring gut microbiota (4). Moreover, it can promote intestinal development in weaning piglets by modulating gut microbiota, improving growth, and reducing diarrhea incidence compared to antibiotics (5). Especially in germ-free mice, *L. plantarum* CCFM8610 affects host health by affecting various pathways and metabolites (6), prevents diet-induced metabolic disorders (7), improves anxiety-like behaviors, and helps alleviate neuropsychiatric disorders (8). Mixtures of *L. casei* and *L. plantarum* have promising anti-allergenic properties (9).

Previous studies have revealed that the administration of *L. plantarum* to pregnant women may possess potential benefits (10, 11). *L. plantarum* 299v could impact gut structure and function in the offspring of rats (10) and may be a tolerable therapy during pregnancy, potentially affecting maternal and neonatal hematological and iron status (11).

**Figure 1:**
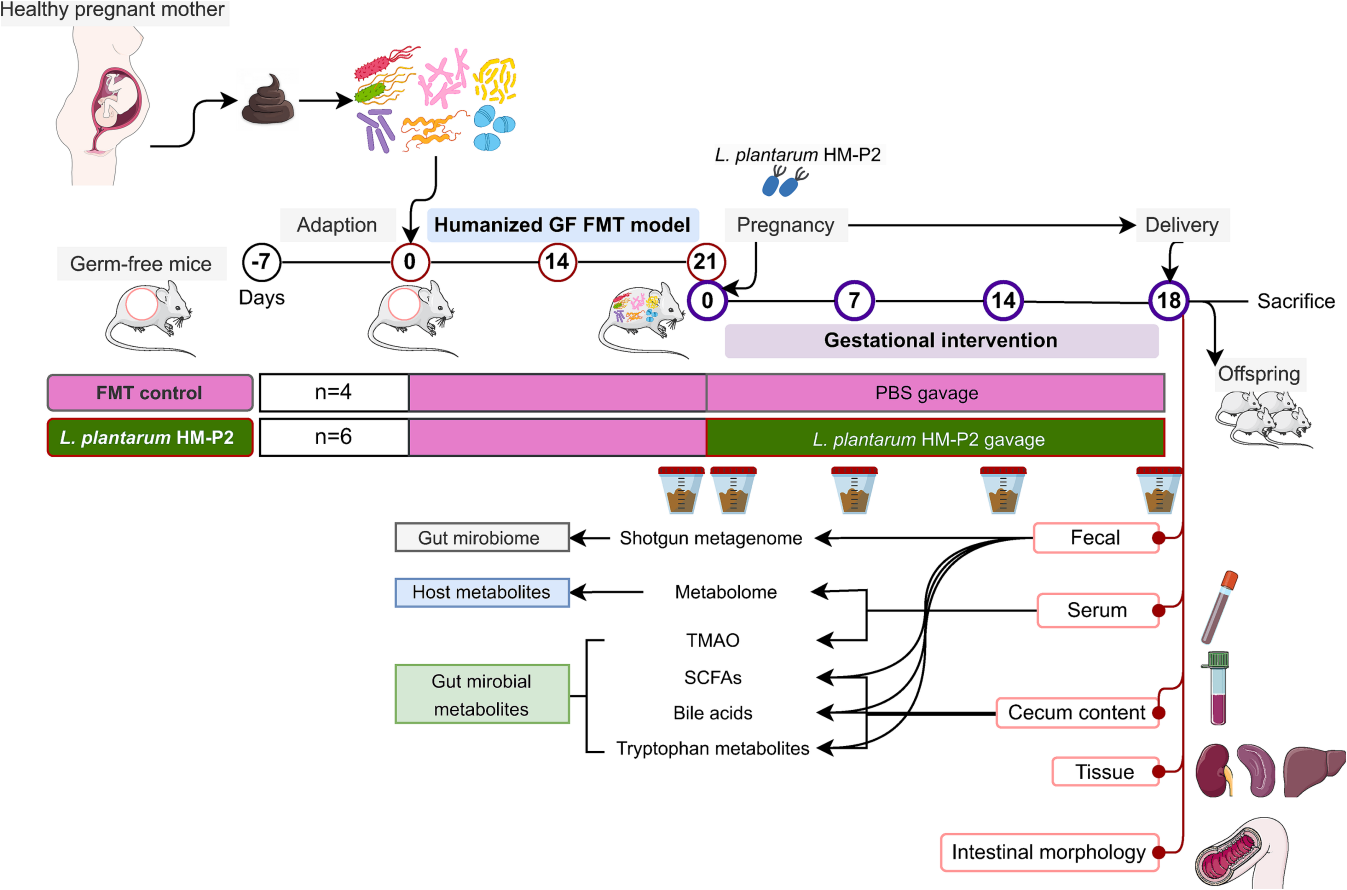
Diagram of experimental design. GF, germ-free; FMT, fecal microbiota transplant; PBS, phosphate buffered saline; TMAO, trimethylamine-N-oxide; SCFAs, short-chain fatty acids.

Although studies have shown that various strains of *L. plantarum* have multiple health benefits for pregnant rats and mice with certain diseases, current evidence is still limited about the effect of probiotics on gestational health. In this study, we combined the fecal microbiome transplant technique with GF mice to create a humanized germ-free mice model with the gut microbiome of pregnant women. We then explored the effects of the *L. plantarum* HM-P2 strain (derived from human breast milk) on gestational mice. The study analyzed the impact of this strain on intestinal morphology, gut microbiome, serum metabolome, immune factors, and gut microbial metabolites in gestational GF mice (Figure 1).

## Materials and methods

### Gestational fecal microbiome mixture preparation

Nine healthy gestational donors were recruited for the study. They were screened and selected based on specific criteria, which included being between 30 and 40 weeks of gestation, not having used antibiotics or any other medicine within the last 3 months, and not experiencing any gastrointestinal symptoms, such as constipation, diarrhea, or discomfort. Stools were collected from these donors for 15 consecutive days and preserved in glycerol prior to freezing. The preparation of the fecal bacterial solution was performed under anaerobic conditions at different phases of fecal microbiome transplant (FMT) and was described in detail previously (12).

### Construction of humanized mice model using fecal microbiome transplant

Germ-free (GF) C57BL/6 J mice (8-week-old, female, *n* = 10, body weight 18–22 g) were obtained from the GemPharmatech Co., Ltd., located in Jiangsu, China. The GF mice were housed in six separate isolators with a 12-h light/dark cycle in a temperature-controlled (22°C ± 1°C) and humidity-controlled (55% ± 10%) room. Humanized GF mouse models were generated by performing fecal microbiome transplant (FMT) twice a week for three consecutive weeks, using a 0.2 mL of fecal sample for each time. In order to prevent environmental microorganisms and operational contamination, all experimental operations were performed under strict aseptic conditions. Subsequently, the models were then evaluated using 16S rRNA sequencing, as described previously in reference (12). Protocols were approved by the Animal Care and Use Committee of GemPharmatech Co., Ltd. (No. GPTAP20220321-1). Experiments were performed following the Guide of Experimental Animals of the Ministry of Science and Technology (Beijing, China).

### Preparation of probiotic *Lactiplantibacillus plantarum* HM-P2

*Lactiplantibacillus plantarum* HM-P2 (Patent No. CN115491329A) was isolated from healthy breast milk, and the strain was stored in the China General Microbiological Culture Collection Center.^1^ Its probiotic properties have been disclosed in our granted patents “CN115491329A” and summarized in Supplementary material S1.

*Lactiplantibacillus plantarum* HM-P2 was selected based on its evaluated probiotic functions. This strain exhibits high bile salt tolerance, enabling it to withstand the high bile salt environment of the human body (such as in the small intestine). It also demonstrates strong inhibitory effects against pathogenic *Escherichia coli* and/or *Staphylococcus aureus*. Therefore, HM-P2 holds promising potential for development and application in the food industry. Furthermore, this strain has received granted both a domestic invention patent in China (CN115491329A) as well as an international PCT (Patent Cooperation Treaty) patent (WO2024060768). Therefore, this study has selected HM-P2 for a detailed investigation into its mechanisms for promoting maternal health during pregnancy.

### Probiotic intervention experiment design

This study aims to determine the direct effects of HM-P2 on maternal health through intervention in humanized pregnant mice. FMT GF mice (*n* = 10) were randomly assigned to two experimental groups, named the control and *L. plantarum* HM-P2 groups. Both groups received standard chow feeding (100 μ L phosphate buffered saline [PBS] solution) and standard chow + *L. plantarum* HM-P2, separately. Probiotic was pre-tested with three doses (low-1*10^8^, middle-1*10^9^, and high-1*10^10^ CFU/mL), and based on *in vitro* safety evaluations and literature references (see Supplementary material S2), the middle dose (1 × 10⁹) was ultimately selected for the FMT experiment. Probiotic was fed at a final dose of 1*10^9^ CFU/mL in PBS solution by oral gavage (2 days/week, p.o., 3 weeks). The GF mice were housed in six separate isolators with a 12-h light/dark cycle in a temperature-controlled (22°C ± 1°C) and humidity-controlled (55% ± 10%) room. The mice were allowed *ad libitum* access to germ-free water throughout the experimental period. Health status was monitored daily throughout the experiment; feces were collected at 0 d, 1 d, 7 d, 14 d, and 18 d of the experiment, and then stored at −80°C for short-chain fatty acids (SCFAs), bile acids, tryptophan analysis, and metagenome analysis. After delivery, the number of offspring was recorded. At the end of the experiment, mice were fasted overnight and sacrificed, and the fresh weight of tissues (total and left liver, spleen, and kidney) was measured. Blood was collected and centrifuged at 3,000 rpm for 10 min and stored in a − 80°C environment until further analysis.

### Evaluation of *Lactiplantibacillus plantarum* HM-P2 colonization

Fecal samples were collected and shotgun metagenomic sequenced to verify the colonization of the *L. plantarum* strain at 0 d, 1 d, 7 d, 14 d, and 18 d.

### Morphological analysis

Small portions of the middle cecum and colon were washed, placed in a 10% phosphate-buffered formalin solution, and stored at room temperature for histological analysis. The evaluated morphological indicators were villi height, top crypt depth, and middle crypt depth. Morphologic analysis was performed on 10 well-oriented and intact villi and 10 crypt foci from each section of the cecum and colon.

### Determination of cytokine and immunoglobulin levels using multiplexed bead-based immunoassays

Multiplexed bead-based immunoassays (Luminex 200 system, Thermo Fisher Scientific, United States) were performed to detect serum levels of cytokines IFN- γ , TGF- β -1, TNF- α , IL-10, IL-8, IL-6, IL-4, TGF- α , IL-22, IL-17, IL1- β , and IL1- α , as well as immune factors such as IgM and IgG, using mice ELISA kits (Cloud-Clone Corp., Katy, TX, United States) according to the manufacturer’s instructions.

### Shotgun metagenomic sequencing

Bacterial genomic DNA was extracted from 100 mg of homogenized fecal samples using the QIAamp DNA Stool Mini Kit (Qiagen, Hilden, Germany) following the manufacturer’s protocol. DNA was examined by 0.8% agarose gel electrophoresis, and the OD value of 260/280 was determined by spectrophotometry. A HiSeq 2,500 (Illumina, CA, United States) with 150 bp paired-end reads was used to perform shotgun metagenomic sequencing of all the DNA samples. FastQC v0.11.9^2^ was used to check the quality of raw sequencing reads. KneadData v0.7.2^3^ was used to trim and remove host sequences. MetaPhlAn2 v2.2.0 (13) was performed for taxonomy annotation and profiling, and HUMAnN2 v0.11.2 (14) was used to perform functional annotation in default settings. After we get the abundances of microbiomes, genes, and metabolic pathways, MEGAHIT v1.2.9 (15) was used to construct metagenome-assembled genomes, Prodigal v2.6.3 (16) was used to predict open reading frames, MetaBAT v2.12.1 (17) was used for genome binning, genome quality was checked by CheckM v1.1.3 (18), and taxonomy was identified by GTDB-Tk v1.0.2 (19).

### Determination of short-chain fatty acids

For fecal samples at 0 d, 1 d, 7 d, 14 d, and 18 d, and cecum content samples at the end of the experiment (18 d), seven SCFAs (acetic acid, butyric acid, caproic acid, propionic acid, isovaleric acid, isobutyric acid, and valeric acid) were determined using a Trace 1,310 gas chromatograph coupled with an ISQ LT (Thermo Fisher Scientific, United States) (20). Briefly, 100 mg of sample was extracted in 50 μL of 15% phosphoric acid with 100 μL of 125 μg/mL 4-methyl valeric acid solution as an internal standard and 400 μL of ether and was centrifuged at 4°C for 10 min at 12000 rpm after vortexing for 1 min, and the supernatant was transferred into the vial before GC–MS analysis. An Agilent HP-INNOWAX (30 m × 0.25 mm ID × 0.25 μm) column was used. Helium was used as the carrier gas at 1 mL/min. Injection was made in split mode at 10:1 with an injection volume of 1 μL and an injector temperature of 250°C. The temperatures of the ion source and interface were 300°C and 250°C, respectively. The column temperature was programmed to increase from an initial temperature of 90°C, followed by an increase to 120°C at 10°C/min, to 150°C at 5°C/min, and finally to 250°C at 25°C/min, which was maintained for 2 min (total run time of 15 min). Mass spectrometric detection of metabolites was performed on ISQ LT (Thermo Fisher Scientific, United States) with electron impact ionization mode. Single ion monitoring (SIM) mode was used with an electron energy of 70 eV. Quantification was made using external standard curves.

### Measurement of tryptophan catabolites

For fecal samples at 0 d, 1 d, 7 d, 14 d, and 18 d, and cecum content samples at the end of the experiment (18 d), 30 tryptophan catabolites were determined using a Vanquish UHPLC (Thermo Fisher Scientific, Haverhill, MA) coupled with ABI Sciex 5,000 mass spectrometer (ABI Sciex, Concord, ON, Canada) according to previous work (21). Briefly, the sample was extracted in 100 μL of 80% methanol aqueous solution, grinding for 60 s, then 900 μL of 10% methanol aqueous solution, grinding for 120 s. Centrifugation was carried out at 12,000 rpm at 4°C for 10 min, and the supernatant was filtered; an equal amount of the internal standard solution was added and then transferred into the vial for analysis. An ACQUITY UPLC HSS T3 column (150 × 2.1 mm, 1.8 μm) (Waters, Milford, MA, United States) was used for chromatographic separation at 40°C. The mobile phase was composed of solvent A (0.1% formic acid in water) and solvent B (0.1% formic acid in methanol). The gradient was: 0 ~ 2 min, 1% B; 2 ~ 3 min, 1 ~ 30% B; 3 ~ 3.5 min, 30% B; 4.5 ~ 8 min, 30 ~ 50% B; 8 ~ 10 min, 50 ~ 95% B; 10 ~ 11 min, 95% B; 11 ~ 17 min, 95 ~ 1% B. The injection volume was 3 μL. The mass parameters were set as the following: 45 arbitrary units (AU), 13 AU, 1 AU, 350°C, and 350°C for sheath gas, aux gas, sweep gas, ion transfer tube, and vaporizer temperature, respectively. The ion source was operated using heated electrospray ionization (ESI) with an ion spray voltage set at 5500 V in positive ion mode. Polarity switching and scheduled selected reaction monitoring (SRM) were employed. Quantification was made using external standard curves.

### Bile acid analysis

For fecal samples at 0 d, 1 d, 7 d, 14 d, and 18 d, and colonic content samples at the end of the experiment (18 d), 39 bile acids were determined using an LC-30 (Shimadzu Corporation, Kyoto, Japan) tandem ABI Sciex 6,500 Plus mass spectrometry (ABI Sciex, Concord, ON, Canada) according to previous work (22). Briefly, the sample was extracted in 400 μL of methanol, vortexing and shaking for 60 s, adding 100 mg of glass beads, and grinding at 55 Hz for 60 s. Repeat the above operation at least twice: ultrasonic at room temperature for 30 min, centrifuged at 12,000 rpm at 4°C for 10 min, and 200 μL of supernatant mixed with 400 μL of water. Take 200 μL of supernatant and add 400 μL of water; vortex for 30 s; take 300 μL of supernatant and filter it through a 0.22 μm membrane; vortex for 30 s. After centrifugation, take 20 μL of supernatant and dilute it 10 times with 180 μL of 30% methanol solution, and add it to the assay vial. An ACQUITY UPLC® BEH C18 column (2.1 × 100 mm, 1.7 μm) (Waters, Milford, MA, United States) was used for chromatographic separation at 40°C. The injection volume was 5 μL. The mobile phase was composed of solvent A (0.01% formic acid in water) and solvent B (100% acetonitrile [ACN]). The gradient was 0 ~ 9 min, 30% B; 9 ~ 14 min, 30 ~ 36% B; 14 ~ 18 min, 36 ~ 38% B; 18 ~ 24 min, 38 ~ 50% B; 24 ~ 32 min, 50 ~ 75% B; 32 ~ 33 min, 75 ~ 90% B; 33 ~ 35.5 min, 90 ~ 30% B. The flow rate was 5 μL/min. The mass parameters were set as the following: the temperature of the ion source was 500°C, curtain gas (CUR) 30 psi, source gas 1 (GAS1) 30 psi, and source gas 2 (GAS2) 35 psi. The ion source was operated using heated electrospray ionization (ESI) with an ion spray voltage set at −4,500 V in negative ion mode. Multiple reaction monitoring (MRM) was employed. Quantification was made using external standard curves.

### Serum choline derivatives

For serum samples at the end of the experiment (18 d), five choline derivatives (choline, betaine, trimethylamine N-oxide [TMAO], creatinine, and L-carnitine) were determined using a Jasper HPLC with SCIEX 4500MD Triple Quadrupole mass spectrometry (ABI Sciex, Concord, ON, Canada) according to previous work (23). Briefly, samples were added to a 2 mL centrifuge tube; 10 μL of internal standard solution, then add 750 μL of 1% formic acid-acetonitrile solution, vortex, centrifuge for 10 min at 12,000 rpm at 4°C, and then resolve and filter the supernatant by 0.22 μm membrane for analysis. An ACQUITY UPLC® BEH HILIC column (2.1 × 100 mm, 1.7 μm) (Waters, Milford, MA, United States) was used for chromatographic separation at 40°C. The injection volume was 5 μL. The mobile phase was composed of solvent A (10 mM ammonium formate) and solvent B (100% ACN). The gradient was 0 ~ 1 min, 80% B; 1 ~ 2 min, 80 ~ 70% B; 2 ~ 2.5 min, 70% B; 2.5 ~ 3 min, 70 ~ 50% B; 3 ~ 3.5 min, 50% B; 3.5 ~ 4 min, 50 ~ 80% B; 4 ~ 6 min, 80% B. The flow rate was 0.4 mL/min. The mass parameters were set as the following: The temperature of the ion source was 500°C, CUR 30 psi, GAS1 5 psi, and GAS2 50 psi. The ion source was operated using heated ESI with an ion spray voltage set at 5000 V in positive ion mode. Multiple reaction monitoring (MRM) was employed. Quantification was made using external standard curves.

### Serum untargeted metabolome analysis

For serum samples at the end of the experiment (18 d), untargeted metabolome was determined using a Vanquish UHPLC System with Orbitrap Exploris 120 MS (Thermo Fisher Scientific, United States) (24). Briefly, samples were thawed at 4°C, vortexed for 1 min, and mixed evenly; then they were transferred into a centrifuge tube and vortexed for 1 min after adding 0.4 mL methanol; then they were centrifuged for 10 min at 12,000 rpm at 4°C, and the supernatant was dried and resolved using 0.15 mL 2-chloro-l-phenylalanine (4 ppm) solution, and then filtered by 0.22 μ m membrane and detected by LC–MS. An ACQUITY UPLC HSS T3 column (150 × 2.1 mm, 1.8 μm) (Waters, Milford, MA, USA) was used for chromatographic separation at 40°C. The flow rate and injection volume were set at 0.25 mL/min and 2 μL, respectively. For LC-ESI^+^-MS analysis, the mobile phases consisted of (B2) 0.1% formic acid in acetonitrile (v/v) and (A2) 0.1% formic acid in water (v/v). Separation was conducted under the following gradient: 0 ~ 1 min, 2% B2; 1 ~ 9 min, 2% ~ 50% B2; 9 ~ 12 min, 50% ~ 98% B2; 12 ~ 13.5 min, 98% B2; 13.5 ~ 14 min, 98% ~ 2% B2; 14 ~ 20 min, 2% B2. For LC-ESI-MS analysis, the analytes were carried out with (B3) acetonitrile and (A3) ammonium formate (5 mM). Separation was conducted under the following gradient: 0 ~ 1 min, 2% B3; 1 ~ 9 min, 2% ~ 50% B3; 9 ~ 12 min, 50% ~ 98% B3; 12 ~ 13.5 min, 98% B3; 13.5 ~ 14 min, 98% ~ 2% B3; 14 ~ 17 min, 2% B3. The MS system with a heated electrospray ionization (ESI) source was operated in both positive and negative ion modes. The parameters were as follows: 3.50 kV and −2.50 kV for ESI^+^ and ESI^−^ source voltage and 325°C capillary temperature. Capillary voltages for negative and positive ionization modes were set at 2500 and 3,500 V, respectively. MS1 range was 100–1,000 m/z, and the number of data-dependent scans per cycle was 4; MS/MS resolving power was 15,000 FWHM, normalized collision energy was 30%, and dynamic exclusion time was automatic.

The raw MS data were first converted to mzXML format by MSConvert in the ProteoWizard software package (v3.0.8789) (25). and processed using XCMS (26) for feature detection, retention time correction, and alignment. The metabolites were identified by accurate mass (< 30 ppm) and MS/MS data, which were matched with HMDB,^4^ Massbank,^5^ LipidMaps,^6^ mzCloud,^7^ and KEGG.^8^

### Statistical analysis

Data were analyzed by R language (Version 4.3.1) (27). Nonparametric tests of independent variables (tissue weights, immune factors, and morphological and SCFAs) between the two groups were performed using the Wilcoxon test.

Relative abundance of gut microbial genera, α -diversity, β -diversity, LEfSe differential analysis, and correlation analysis were performed in the R library “microeco” (version 0.5.1) (28). Functional profiling of metagenomes was analyzed using MicrobiomeAnalyst 2.0 (29).^9^ Untargeted metabolome, SCFAs, bile acids, and tryptophan were analyzed and visualized for differential analysis and PLS-DA, and enrichment analysis of targeted metabolites was performed in MetaboAnalyst 5.0 (30).^10^ Only metabolites present in >50% of the samples were kept for further analysis and then log2 normalized and Pareto-scaled before further statistical analysis. Association analysis of microbial abundance, contents of SCFA, bile acids, tryptophan metabolites, and morphological indexes was performed and visualized in the Hmisc package and heatmap package in R using Spearman rank correlation. All statistical analyses were considered significant at an adjusted *p* value less than 0.05 level unless specifically noted.

Diagrams of the experiment design and mechanism were visualized using icons from Bioicons^11^ and Flaticon,^12^ and graphs were paneled using Inkscape 1.3.^13^

## Results

### Colonization of *Lactiplantibacillus plantarum* HM-P2 in humanized GF mice

**Figure 2:**
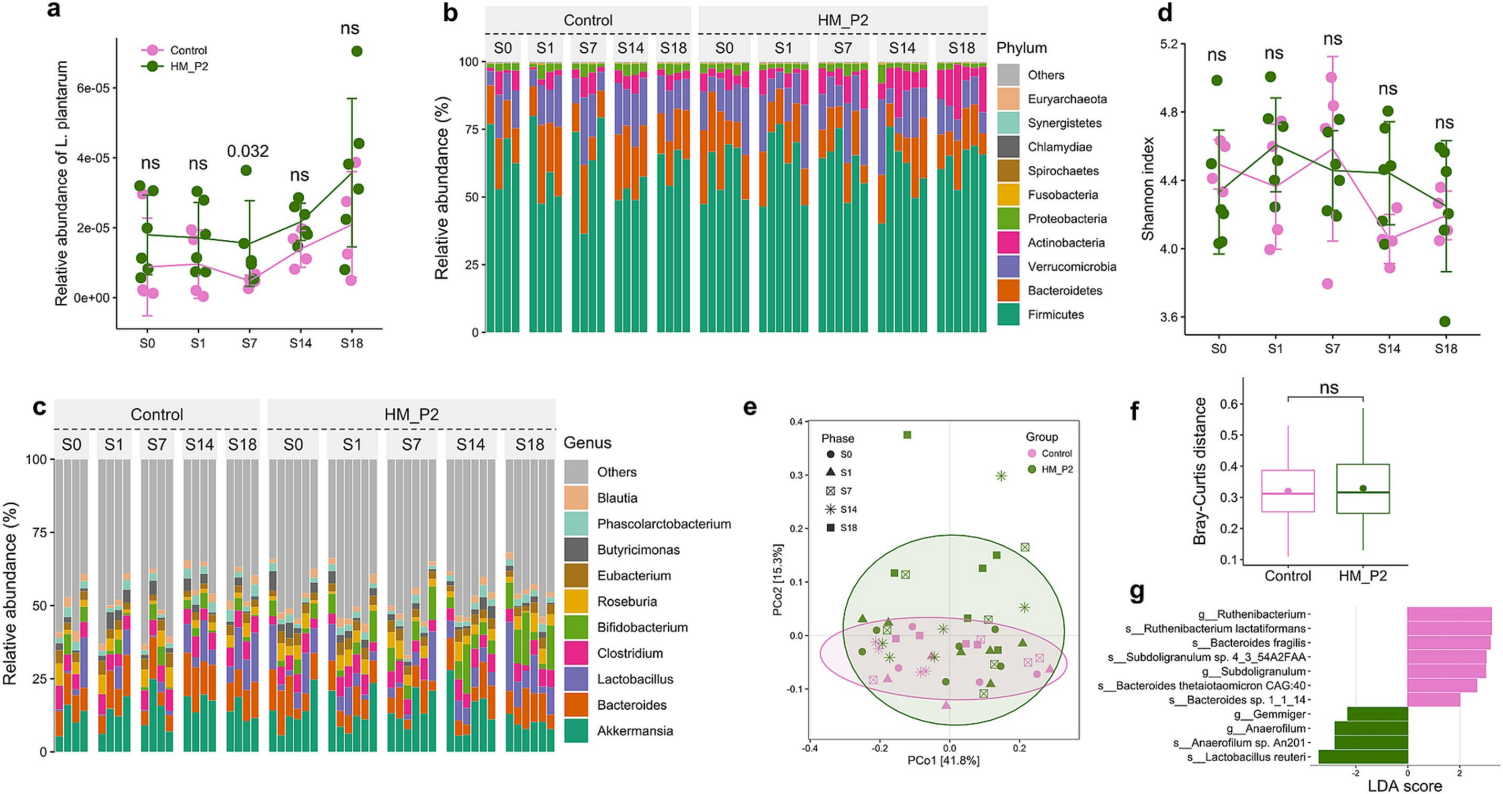
Effects of *L. plantarum* HM-P2 administration on gut metagenome of GF mice at 0, 1, 7, 14, and 18 d of gestation. **(A)** Relative abundance of *L. plantarum*. **(B)** Composition and changes of gut microbiome at phylum level. **(C)** Composition and changes of gut microbiome at the genus level. **(D)** Change of α-diversity (Shannon) index. **(E)** Principal coordinate analysis (PCoA) plot. **(F)** β-diversity index (Bray-Curtis distance). **(G)** Significant difference in microbes by linear discriminant analysis Effect Size. *p*-values of the Wilcoxon tests are labeled. “ns,” not significant (at *p* < 0.05 level).

*Lactiplantibacillus plantarum* HM-P2 treatment significantly increased (*p* = 0.032) the relative abundance of fecal *L. plantarum* of mice at 7 d of gestation, compared with the control group (Figure 2A). This showed that *L. plantarum* HM-P2 successfully colonized the gut of GF mice at this stage of gestation.

### *Lactiplantibacillus plantarum* HM-P2 administration altered fecal metagenome composition and pathway

Germ-free mice were fecal microbiota transplanted with stool from healthy pregnant women and were fed a diet supplemented with *L. plantarum* HM-P2 from gestation to delivery (0–18 d). The composition and dynamic changes of the stool microbiome after HM-P2 treatment were studied using shotgun metagenomic sequencing. A total of 13,636,188,396 bp raw sequences were obtained from 50 samples. The average raw data of 272,723,767.9 bp was obtained, and then the data were assigned to 5 kingdoms, 104 phyla, 85 classes, 163 orders, 348 families, 1,315 genera, and 5,786 species.

Composition analysis showed that Firmicutes (~50%), Bacteroids (~20%), Verrucomicrobia (~15%), Proteobacteria (<5%), Actinobacteria (<5%), Fusobacteria, and so on, were the dominant phyla in all samples (Figure 2B). At the genus level, *Akkermansia*, *Bacteroids*, *Lactobacillus*, *Clostridium*, *Bifidobacterium*, *Roseburia*, *Eubacterium*, *Phascolarctobacterium*, *Butyricimonas,* and *Blautia* were the dominant genera (Figure 2C).

𝛼-diversity indexes (Shannon) from 0 to 18 d of gestation showed that no significant difference was found between the HM-P2 and control groups (Figure 2D). Principal coordinate analysis (PCoA) showed that there was a moderate separation of the HM-P2 group and control group, and PC1 and PC2 together explained 57.1% of the total difference (Figure 2E). Bray-Curtis distance was not significantly different between the HM-P2 group and the control group (Figure 2F).

Differential analysis by linear discriminant analysis Effect Size (LefSe) (“Group” as the main effect and “Phase” as the sub-effect) showed that *L. reuteri*, *Anaerofilum* sp. *An201*, and *Gemmiger* were significantly higher (adjusted *p* < 0.05) in the HM-P2 group, while *Bacteroides thetaiotaomicron* sp. *CAG:40*, *Bacteroides* sp. 1_1_14, *Bacteroides fragilis*, *Subdoligranulum* sp. 4_3_54A2FAA, and *Ruthenibacterium lactatiformans* were significantly higher in the control group (adjusted *p* < 0.05) (Figure 2G).

A total of 89 metagenomic genes were differentially expressed in the HM-P2 group compared with the control group at 18 d. These differentially expressed genes were significantly (p < 0.05) enriched in starch and sucrose metabolism (see Supplementary Table S1).

### *Lactiplantibacillus plantarum* HM-P2 treatment altered colon and cecum morphology

**Figure 3:**
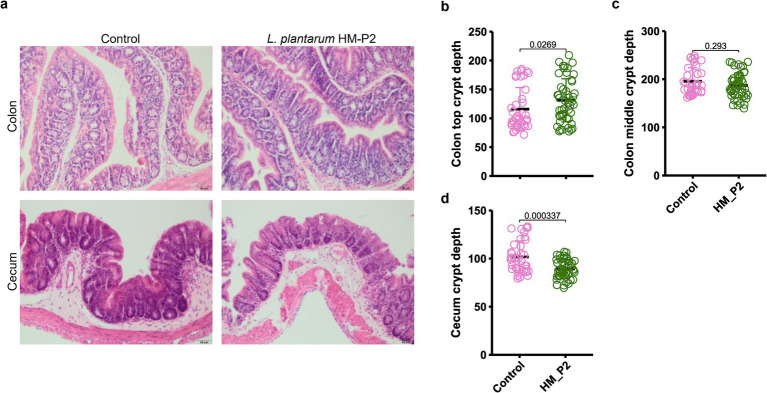
Effects of *L. plantarum* HM-P2 treatment on intestinal morphology. **(A)** Hematoxylin and eosin (H&E) images of the cecum and colon of GF mice. Bars = 50 μm. **(B)** Colon top crypt depth. **(C)** Colon middle crypt depth. **(D)** Cecum crypt depth. *p*-values of the Wilcoxon tests are labeled. “ns,” not significant (at *p* < 0.05 level).

The influence of human breast milk-derived *L. plantarum* HM-P2 on the morphometric indices of the cecum and colon was measured (Figure 3A). In the colon, top crypt depth was significantly higher in the HM-P2 group than the control group (Figure 3B); colon middle crypt depth showed no significant difference between the two groups (Figure 3C). In the cecum, crypt depth was significantly higher in the control group compared with the HM-P2 group (Figure 3D).

### *Lactiplantibacillus plantarum* HM-P2 administration changed microbial bile acids and tryptophan metabolites in fecal

**Figure 4:**
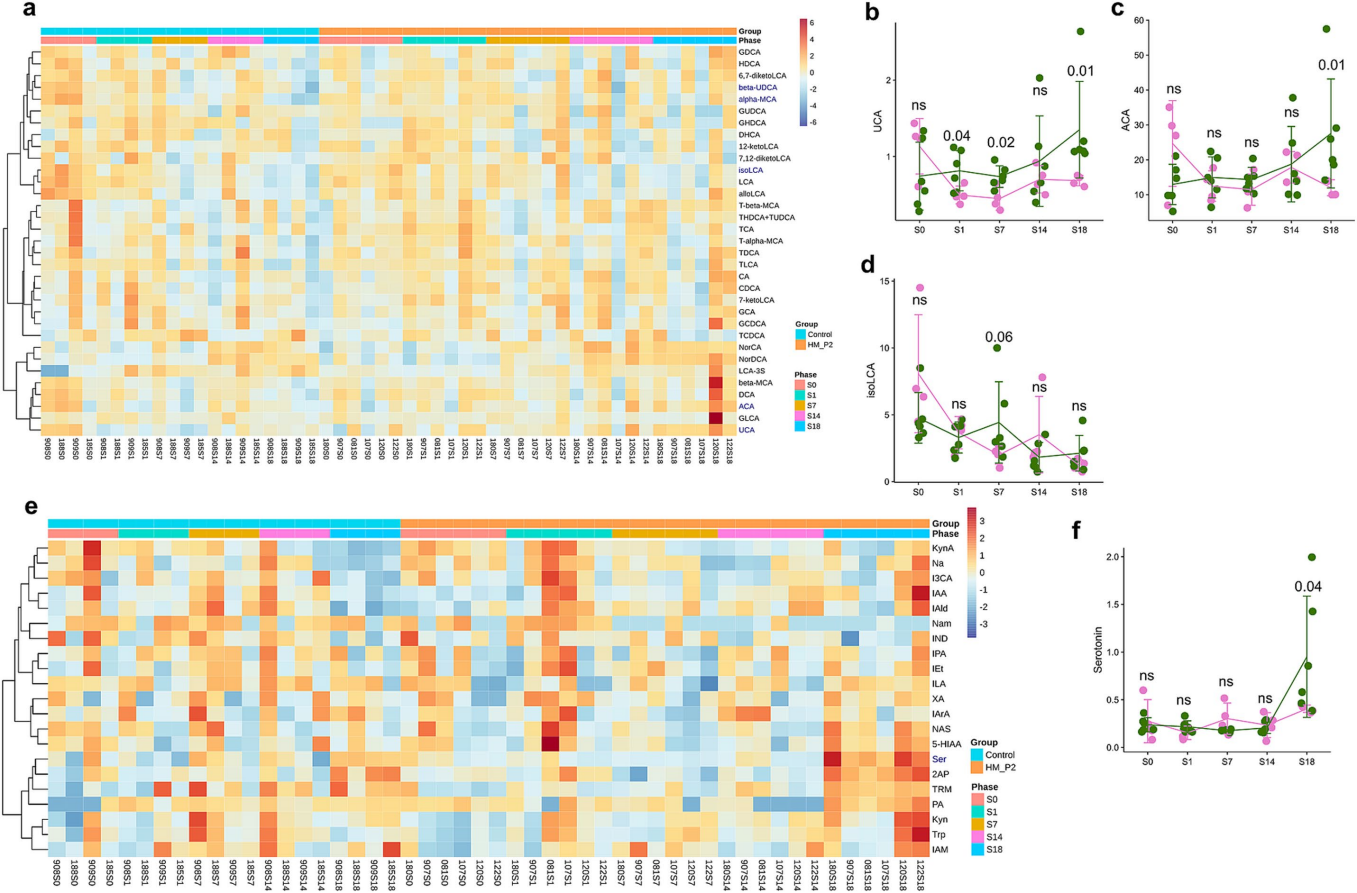
Effects of *L. plantarum* HM-P2 administration on fecal metabolites of GF mice at 0, 1, 7, 14, and 18 d of gestation. **(A)** Heatmap of microbial bile acids. **(B)** Ursocholic acid (UCA). **(C)** Allo-cholic acid (ACA). **(D)** Isolithocholic acid (isoLCA). **(E)** Heatmap of microbial tryptophans. **(F)** Serotonin. *p*-values of the Wilcoxon tests are labeled. “ns,” not significant (at *p* < 0.05 level).

In fecal samples, the contents and changes of microbial bile acids are shown in Figure 4A. Among them, the contents of primary bile acids (unconjugated, ursocholic acid [UCA; Figure 4B), allo-cholic acid (ACA; Figure 4C)] and secondary bile acids [unconjugated, isolithocholic acid (isoLCA; Figure 4D)] showed significant differences between the HM-P2 and control groups. Other bile acids were not significantly different between the two groups.

In fecal samples, the contents and changes of 21 tryptophan metabolites are shown in Figure 4E. Among them, the concentration of serotonin (5-hydroxytryptamine, 5-HT) revealed a significant difference between the HM-P2 and control groups (Figure 4F). The other tryptophan metabolites were not significant between the two groups.

### *Lactiplantibacillus plantarum* HM-P2 administration altered microbial short-chain fatty acids and bile acids in cecum contents

**Figure 5:**
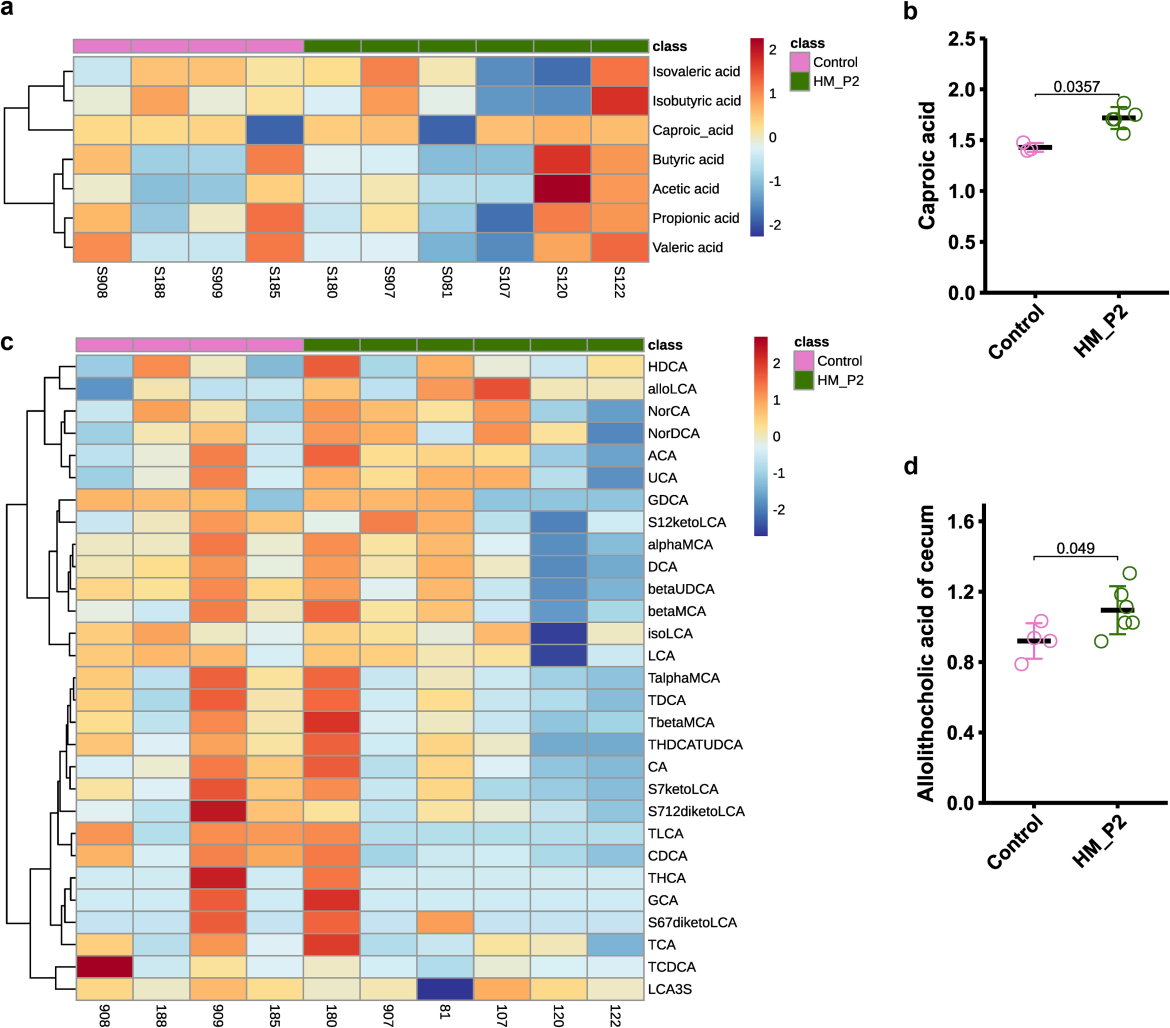
Effects of *L. plantarum* HM-P2 administration on metabolites of cecum contents of GF mice at 18 d of gestation. **(A)** Heatmap of SCFA. **(B)** Caproic acid. **(C)** Heatmap of bile acids. **(D)** Allolithocholic acid. *p*-values of the Wilcoxon tests are labeled. “ns,” not significant (at *p* < 0.05 level).

The contents of short-chain fatty acids (SCFAs) in cecum contents are shown in Figure 5A. Compared to the mice in the control group, caproic acid significantly increased in the HM-P2 group at 18 d of gestation (Figure 5B), and other SCFAs (acetic acid, propionic acid, isovaleric acid, valeric acid, and isobutyric acid) showed no significant difference between the two groups at 18 d of gestation.

The contents of bile acids in the cecum contents are shown in Figure 5C. Among 30 identified bile acids, allolithocholic acid was significantly higher in the HM-P2 group at 18 d of gestation compared with the control group (Figure 5D). Other bile acids were not significantly different between the two groups.

### *Lactiplantibacillus plantarum* HM-P2 administration changed serum metabolome

**Figure 6:**
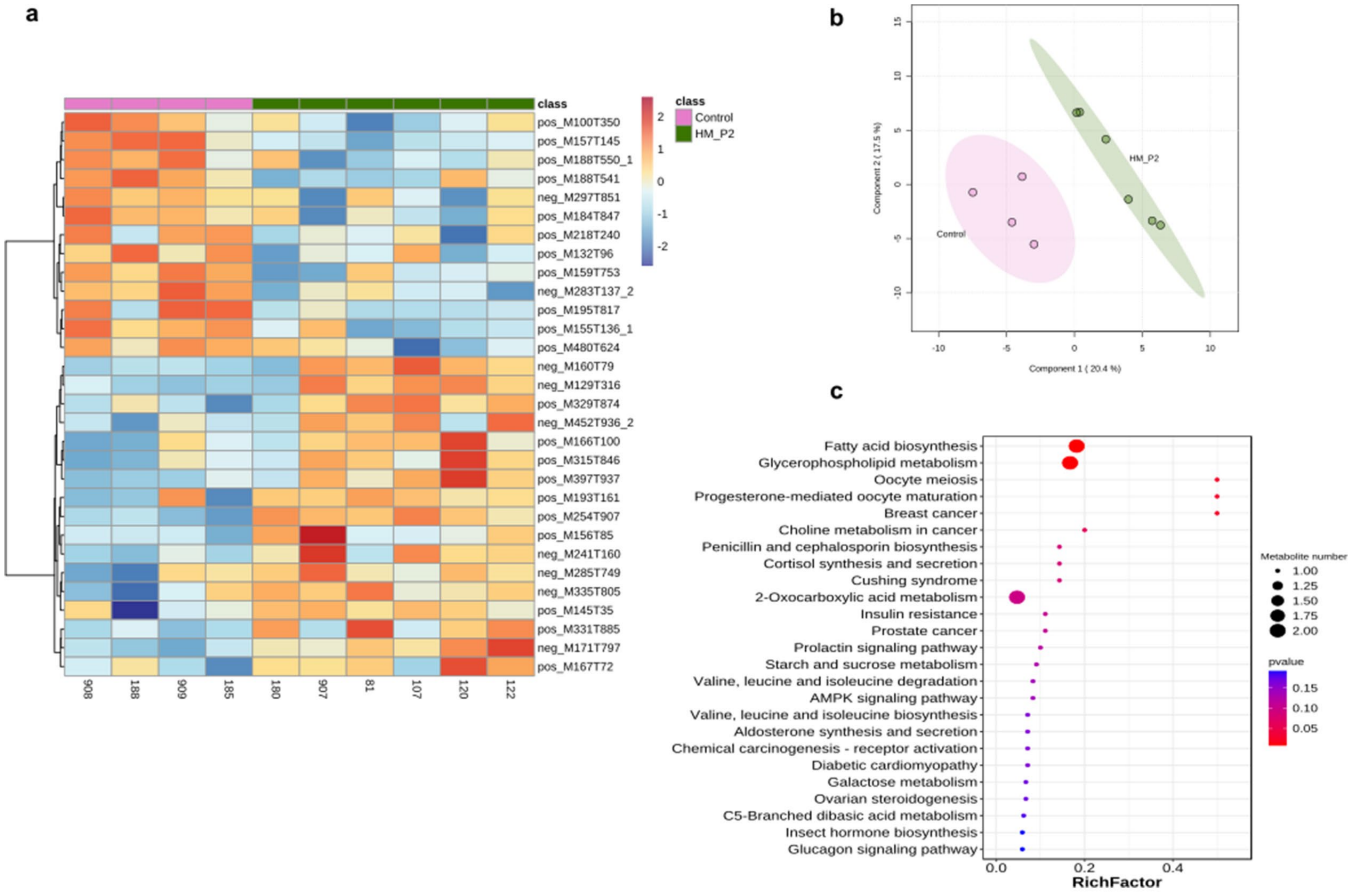
Effects of *L. plantarum* HM-P2 administration on serum metabolites of GF mice at 18 d of gestation. **(A)** Heatmap of top-30 significantly different metabolites of both positive and negative modes. **(B)** Sparse partial least-squares discriminant analysis scores plot. **(C)** KEGG enrichment dot plot of significantly different metabolites. The size of the dots represents the number of metabolites, and the color intensity indicates the *p*-value.

Untargeted metabolites of serum samples at 18 d of gestation were analyzed in both positive and negative modes. A total of 448 (287 positive modes and 161 negative modes) serum metabolites were identified and quantified, with the top 30 most abundant metabolites presented in Figure 6A. Sparse partial least-squares discriminant analysis (sPLS-DA) showed that there were clear differences between these two groups (Figure 6B). There were 17 positive and 6 negative metabolites significantly different in the control and HM-P2 groups. Enrichment analysis of significant difference metabolites showed that they were enriched mainly in fatty acid biosynthesis, glycerophospholipid metabolism (Figure 6C).

In addition, compared with the control group, oral administration of *L. plantarum* HM-P2 during gestation of humanized GF mice showed no significant differences on the offspring numbers (See Supplementary Figure S1), and the fresh weight of five types of tissues (liver, left lobe of liver, left and right kidney, and spleen; See Supplementary Table S2), and SCFAs of fecal (See Supplementary Figure S2), and tryptophan of cecum contents (See Supplementary Figure S3), and serum immune factors, such as immunoglobulin M (IgM), and immunoglobulin G (IgG), and inflammatory cytokine factors, such as interferon-gamma (IFN-*γ*), transforming growth factor-beta-1 (TGF-*β*-1), tumor necrosis factor alpha (TNF-*α*), interleukin-10 (IL-10), interleukin-8 (IL-8), interleukin-6 (IL-6), interleukin-4 (IL-4), transforming growth factor-alpha TGF-α, interleukin-22 (IL-22), interleukin-17 (IL-17), interleukin 1-beta (IL1-β), interleukin 1-alpha (IL1-α; See Supplementary Table S3), and the serum choline derivatives (choline, trimethylamine N-oxide (TMAO), betaine, creatinine, and carnitine; See Supplementary Figure S4).

### SCFA-encoding genes were associated with SCFA contents

**Figure 7:**
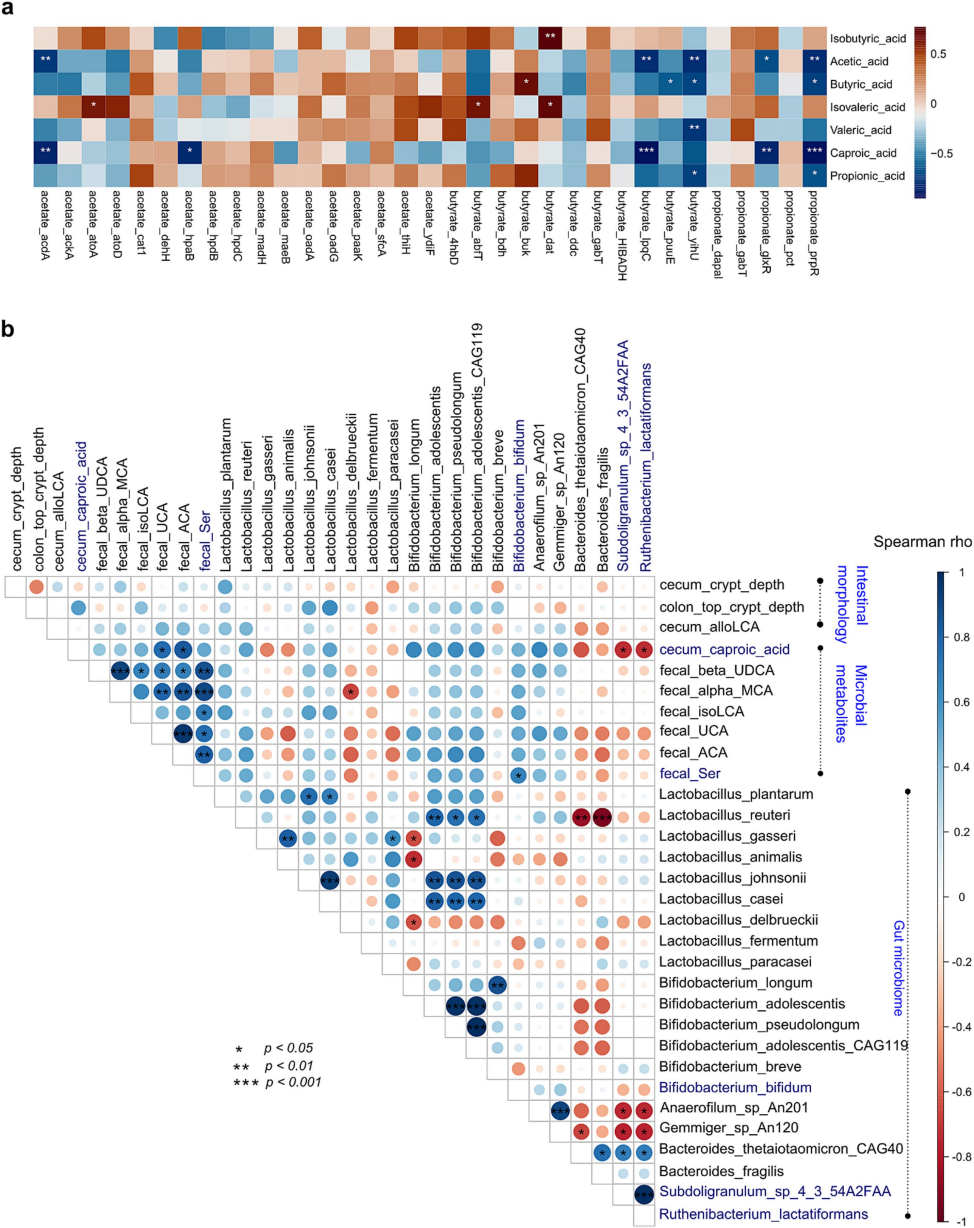
Association analysis of intestinal morphology, microbes, microbial genes, and microbial metabolites. **(A)** Spearman correlation and heatmap of gut microbial SCFA-biosynthesis genes and fecal SCFAs. **(B)** Spearman correlation and heatmap of the gut microbiome with intestinal morphology and microbial metabolites. Cell color represents values of Spearman rho. *, *p* < 0.05; **, *p* < 0.01; and ***, *p* < 0.001.

Spearman correlation showed that microbial genes encoding SCFA biosynthesis were significantly correlated with SCFA contents. Fecal microbial acyl-CoA dehydrogenase (*acdA*) was negatively correlated with the content of fecal acetic acid, butyrate kinase (*buk*) was positively correlated with the content of fecal butyric acid (*p* < 0.05, rho = 0.685) and isobutyric acid (*p* < 0.05, rho = 0.77), while 3-sulfolactaldehyde reductase (*yihU*) showed an opposite relationship (*p* < 0.05, rho = −0.72) with butyric acid; propionate catabolism operon regulatory protein (*prpR*) was negatively correlated with the content of fecal propionic acid (*p* < 0.05, rho = −0.673; Figure 7A).

### Association of gut microbiome, microbial metabolites, and intestinal morphometric indices

To further confirm probiotic effects on gut microbiome, microbial metabolites, and intestinal morphometric indices, Spearman correlation analysis was performed. Results showed that gut microbes were associated with those significantly different bile acids and tryptophans. *Subdoligranulum* sp. 4–3-54A2FAA and *Ruthenibacterium lactatiformans* were negatively correlated with cecum caproic acid (*p* < 0.05, both rho = −0.74), and *B. bifidum* was positively correlated with fecal serotonin (*p* < 0.05, rho = 0.48; Figure 7B).

## Discussion

**Figure 8:**
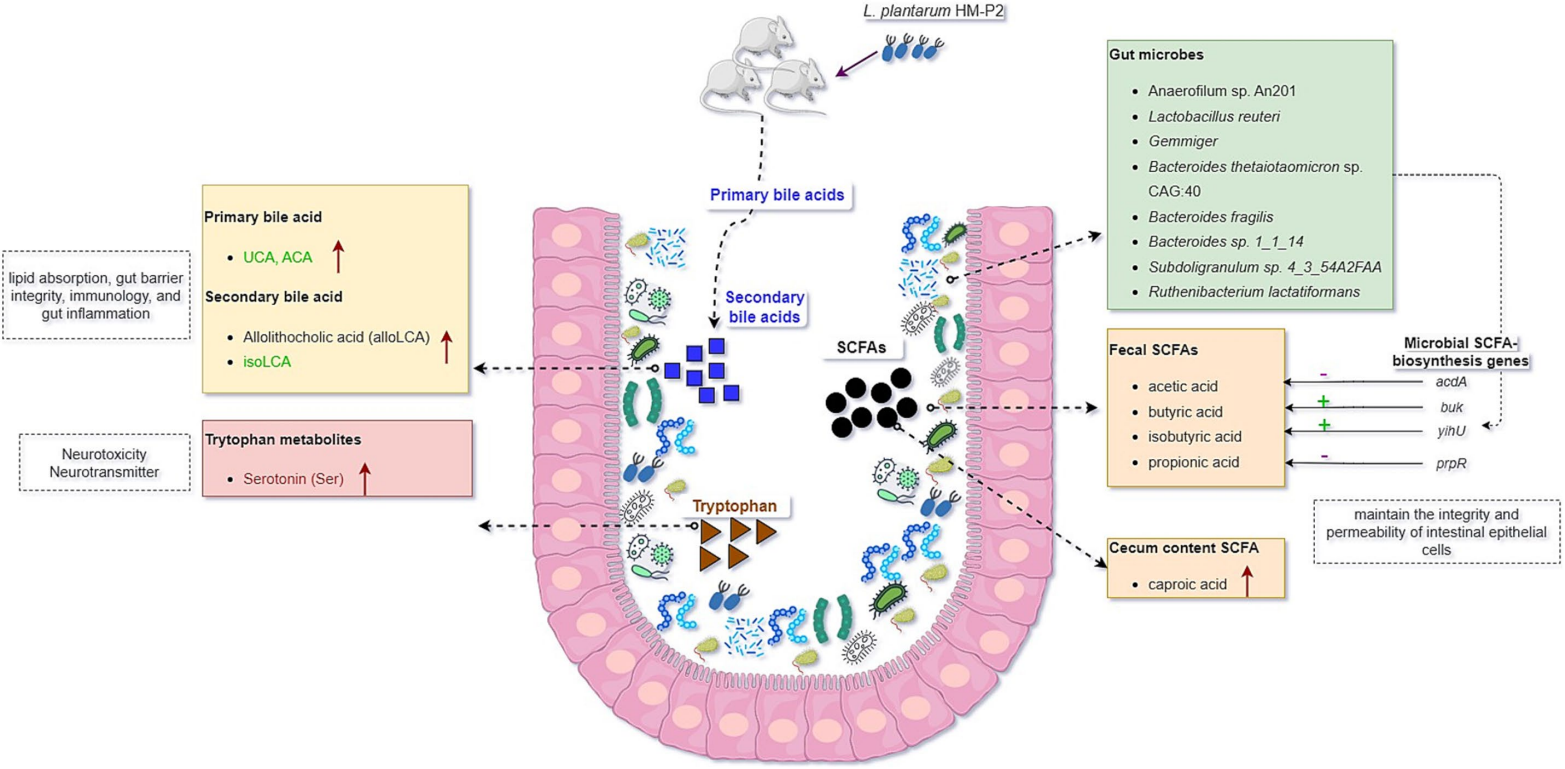
Graphical summary of the effects of oral administration of human milk probiotic *L. plantarum* HM-P2 on gestational health of humanized GF mice.

The experiment investigating the effects of *L. plantarum* HM-P2 on the intestinal morphology, microbiome, serum metabolome, immune factors, and gut microbial metabolites of gestational humanized germ-free mice has provided valuable insights into the potential health benefits of probiotic administration during pregnancy. The results of this study contribute to our understanding of the effects of *L. plantarum* HM-P2 on the microbial metabolism and health of pregnant mice (Figure 8). The results of the experiment revealed several significant outcomes.

Firstly, the administration of *L. plantarum* HM-P2 led to alterations in the gut morphology and gut microbiota composition, promoting the top crypt depth of the colon and the abundance of *L. reuteri*, *Anaerofilum* sp. An201 (Ruminococcaceae family), and *Gemmiger* of gestational GF mice. This effect has also been reported for other *Lactobacillus* species. For example, *L. paracasei* has also been shown to decrease crypt depth in the gut, which is associated with improved gut morphology and barrier function. *L. rhamnosus* GG supplementation increased the ratio of villus height to crypt depth, indicating a positive effect on gut morphology (31). *L. reuteri* is a commensal microbe in the gut of humans and animals, and it could modulate the microbial composition and diversity of preterm infants and promote the development of the piglets’ intestinal mucosal system (32, 33). Gemmiger is a butyrate-producing microbe with anti-inflammatory and mucosal barrier-maintaining properties (34). Several other strains of *L. plantarum* have been shown to significantly influence the intestinal microbiome. *L. plantarum* FZU3013 could make structural changes in the intestinal microbiome of the mice, in particular by modulating the relative abundance of some function-related microbial phylotypes (35). LP9010 reorganized the gut microbiome by increasing the relative abundance of Bacteroidetes and Firmicutes and decreasing the relative abundance of Proteobacteria and Verrucomicrobia (36). Lp082 improved the biological barrier by increasing the diversity, optimizing the species composition and the structure of the gut microbiota, and increasing bacteria production of SCFAs. *L. plantarum* DP189 reshaped the gut microbiota in Parkinson’s disease mice by reducing the number of pathogenic bacteria (Proteobacteria and Actinobacteria) and increasing the abundance of probiotics (*Lactobacillus* and *Prevotella*) (37). However, other strains showed no significant effects on gut microbiome. Oral administration of *L. plantarum* over 14 days did not change the gut microbiota composition, indicated by no significant difference in *α* and *β* diversities (38). Also, the digestion of *L. plantarum* resulted in higher levels of *Lactobacillus* in the digestive tract but did not result in global alterations to the intestinal microbiome (39). These changes in the microbiome are indicative of the probiotic’s ability to modulate the gut environment and promote a more favorable microbial balance.

SCFAs are the primary constituents of the intestinal tract and are important metabolites of gut microbiota, but their impact on metabolism during pregnancy is still less reported. Numerous studies have demonstrated the immunomodulatory properties of SCFAs, as well as their ability to lower pro-inflammatory factor expression and the inflammatory response. Administration of *L. plantarum* HM-P2 could increase caproic acid in the cecum of GF mice. Previous studies showed that the content of propionic and linear caproic acids might be a crucial factor in maintaining lower anthropometric measurements during pregnancy (40). SCFAs can protect the intestinal epithelial barrier, encourage mucin secretion in goblet cells, and preserve the integrity and permeability of intestinal epithelial cells (41). This implied that *L. plantarum* HM-P2 could potentially improve intestinal health by modulating the contents of caproic acid during pregnancy.

Bile acids are crucial signaling molecules that have a tight connection to the metabolism of cholesterol. Several studies showed that the biotransformation of primary bile acids by intestinal bacteria can decrease blood lipid levels and prevent fat accumulation in the liver (42). Previous studies have shown that several probiotic *L. plantarum* strains, such as HAC01, could alleviate hyperglycemia and type 2 diabetes mellitus by regulating glucose metabolism in the liver. *L. plantarum* HAC01 led to an increase in butyric acid, which could have a beneficial effect in the diabetic model (43). *L. plantarum* H-87 could inhibit liver fat deposition, insulin resistance, and lipid digestion by changing bile acid enterohepatic circulation and eventually alleviate high-fat-diet-induced obesity (44). *L. plantarum* 69–2 and galactooligosaccharides could activate the hepatic AMP-activated protein kinase and the histone/protein deacetylase SIRT1 signaling pathway by regulating the gut microbiota and metabolites through the liver-gut axis to restore the hepatic antioxidant activity to alleviate aging (45). In this study, administration of *L. plantarum* HM-P2 could increase fecal primary bile acids and secondary bile acids both in the cecum and fecal, showing their potential benefits in regulating host lipid metabolism.

Tryptophan is obtained mainly from the diet and is necessary to the human body. Current evidence supports the fact that tryptophan metabolite derived from gut microbiota is essential for the mucosal immune system. In this study, the administration of *L. plantarum* HM-P2 could also increase fecal serotonin. Serotonin is a broadly distributed neurotransmitter in brain regions and affects a variety of functions, including affective mood, impulsivity, learning and memory, attention, sleep, aggression, and neurovegetative control (46). Serotonin showed increased concentration in pregnant women, and it plays an important role in maintaining pregnancy and promoting the health of newborns (47). Previous studies also showed that *L. plantarum* LP9010 supplementation inhibited neuroinflammation by up-regulating the levels of neurotransmitters, especially serotonin, niacin, and 5-hydroxyindole acetic acid (36). The level of indole-3-acetic acid, an important tryptophan metabolite in the liver, serum, and colon, was elevated after *Lactobacillus* + Trp treatment (48), and indole-3-lactic acid in *L. plantarum* DPUL-S164-TM plays a key role in improving intestinal barrier function and alleviating inflammation (49). Probiotics can promote the synthesis of neurotransmitters such as serotonin precursor tryptophan and gamma-aminobutyric acid (GABA), thereby contributing to emotional stability. Low levels of tryptophan can lead to reduced serotonin and GABA production, increasing the risk of depression (50). All the pieces of evidence above indicate the potential gestational benefits of HM-P2 in maintaining pregnancy and promoting neonatal health.

TMAO, a typical uremic toxin, is related to the consumption of quaternary amines, which are well-known bacterial osmoprotectants and are frequently found in fruits, vegetables, meat, and seafood. These amines include betaine, choline, and L-carnitine, and they are highly correlated with the risks of atherosclerosis and cardiovascular disease. Although no significance was found in this study, other *L. plantarum* strains have been found to reduce serum TMAO levels in mice challenged with choline, with *L. plantarum* LP1145 showing a significant effect (51).

Furthermore, although several *L. plantarum* strains have been shown to have anti-inflammatory effects, this experiment showed that *L. plantarum* HM-P2 did not influence immune factors. Oral administration of *L. plantarum*-12 down-regulated pro-inflammatory factors tumor necrosis factor-*α*, IL-8, and IL-1β levels and up-regulated anti-inflammatory factor IL-10 level of mice (52). *L. plantarum* DP189 increased the levels of superoxide dismutase, glutathione peroxide, and IL-10 and decreased the levels of malondialdehyde, reactive oxygen species, TNF-α, IL-6, and IL-1β (37). Lp082 optimized the immune barrier by reducing the content of IL-1β, IL-6, TNF-α, myeloperoxidase, and IFN-*γ* and increasing IL-10, TGF-β1, and TGF-β2, playing a protective role by protecting the intestinal mucosal barrier, attenuating the inflammatory response, and regulating microbial imbalance (53).

*L. plantarum* has been shown to significantly influence the serum metabolome of germ-free mice. Huang et al. (6) identified key metabolites affected by *L. plantarum* CCFM8610, including L-methionine, D-tryptophan, indoleacrylic acid, DL-acetylcarnitine, and L-norleucine. This was further supported by Marco et al. (3), who found that *L. plantarum* adapts to the gut habitat by upregulating genes involved in carbohydrate transport and metabolism. *L. plantarum* also showed regulatory effects on glucose and lipid metabolism (54, 55). This study also showed that *L. plantarum* HM-P2 impacted the serum metabolome in several metabolic pathways, such as phosphonate and phosphinate metabolism, valine, leucine, and isoleucine biosynthesis, glycerophospholipid metabolism, and fatty acid biosynthesis, suggesting that *L. plantarum* HM-P2 may have broader systemic effects beyond the gut, potentially influencing overall metabolic health.

Probiotic supplementation during pregnancy has been shown to have potential clinical benefits for both the mother and the baby. It may reduce the risk of preterm delivery, perinatal infections, and functional gastrointestinal diseases (1); increase the levels of immune markers in cord blood and breast milk (56); help restore gut microbiota balance (57); and may reduce the risk of metabolic syndrome in the future (58). Although research on the effects of *L. plantarum* probiotic supplementation during pregnancy in mice is limited, studies on other probiotic strains have shown potential benefits. For example, *L. rhamnosus GG* and *L. gasseri K7* supplementation during pregnancy and lactation in mice can modulate the microbiota of the mesenteric lymph nodes and mammary gland (59), maternal administration of *L. acidophilus* and *B. infantis* probiotics can promote gut development in mouse offspring (60), and *L. fermentum* CECT5716 supplementation during pregnancy and lactation in rats can impact the lipid profile, immune system, and microbiota of both the mother and offspring (61). These studies collectively suggest that probiotic supplementation during pregnancy can have beneficial effects, including its neuroprotective and cognitive health-promoting effects, but more research is needed to specifically investigate the effects of *L. plantarum*.

### Limitations of the study

Due to the limited number of GF mice used in this study, the effects of *L. plantarum* HM-P2 on the offspring’s health were not investigated. Future studies may include examining the effects of *L. plantarum* HM-P2 on offspring gut microbial metabolism and health of humanized GF mice. Additionally, we may increase the sample size and explore the effects of *L. plantarum* HM-P2 in clinically controlled trials of gestational and gastrointestinal diseases such as gestational diabetes mellitus (GDM) and irritable bowel syndrome, functional gastrointestinal disorders, and necrotizing enterocolitis.

## Data Availability

The raw sequence metagenome data reported in this paper have been deposited in the Genome Sequence Archive^41^ in the National Genomics Data Center^42^, China National Center for Bioinformation/Beijing Institute of Genomics, Chinese Academy of Sciences (GSA: CRA014952), which are publicly accessible at https://ngdc.cncb.ac.cn/gsa. Accession numbers are listed in the key resources table. *The metabolomic data* is available at the NIH Common Fund’s National Metabolomics Data Repository (NMDR) website, the Metabolomics Workbench (https://www.metabolomicsworkbench.org), where it has been assigned Project ID PR001923. The data can be accessed directly via its project DOI: https://doi.org/10.21228/M85149. This work is supported by Metabolomics Workbench/National Metabolomics Data Repository (NMDR) (Grant No. U2C-DK119886), Common Fund Data Ecosystem (CFDE) (Grant No. 3OT2OD030544), and Metabolomics Consortium Coordinating Center (M3C) (Grant No. 1U2C-DK119889). Any additional information required to reanalyze the data reported in this paper is available from the lead contact upon request.

## Footnotes

^1^CGMCC, https://cgmcc.net/, No. 23651.

^2^ https://www.bioinformatics.babraham.ac.uk/projects/fastqc

^3^ https://bitbucket.org/biobakery/kneaddata

^4^ http://www.hmdb.ca

^5^ http://www.massbank.jp/

^6^ http://www.lipidmaps.org

^7^ https://www.mzcloud.org

^8^ http://www.genome.jp/kegg/

^9^ https://www.microbiomeanalyst.ca/

^10^ https://www.metaboanalyst.ca/

^11^ https://bioicons.com

^12^ https://www.flaticon.com

^13^ https://inkscape.org

## References

[1] Baldassarre M Palladino V Amoruso A Pindinelli S Mastromarino P Fanelli M. Rationale of probiotic supplementation during pregnancy and neonatal period. Nutrients. (2018) 10:1693. doi: 10.3390/nu10111693 30404227 PMC6267579

[2] Guidone A Zotta T Ross RP Stanton C Rea MC Parente E. Functional properties of *Lactobacillus plantarum* strains: a multivariate screening study. LWT Food Sci Technol. (2014) 56:69–76. doi: 10.1016/j.lwt.2013.10.036

[3] Marco ML Peters THF Bongers RS Molenaar D van Hemert S Sonnenburg JL. Lifestyle of *Lactobacillus plantarum* in the mouse caecum. Environ Microbiol. (2009) 11:2747–57. doi: 10.1111/j.1462-2920.2009.02001.x, PMID: 19638173 PMC2978903

[4] Sun M Liu Y Song Y Gao Y Zhao F Luo Y. The ameliorative effect of *Lactobacillus plantarum* −12 on DSS-induced murine colitis. Food Funct. (2020) 11:5205–22. doi: 10.1039/D0FO00007H, PMID: 32458908

[5] Wang T Teng K Liu Y Shi W Zhang J Dong E. *Lactobacillus plantarum* PFM 105 promotes intestinal development through modulation of gut microbiota in weaning piglets. Front Microbiol. (2019) 10:90. doi: 10.3389/fmicb.2019.00090, PMID: 30804899 PMC6371750

[6] Huang P Yi S Yu L Tian F Zhao J Zhang H. Integrative analysis of the metabolome and transcriptome reveals the influence of *Lactobacillus plantarum* CCFM8610 on germ-free mice. Food Funct. (2023) 14:388–98. doi: 10.1039/D2FO03117E 36511852

[7] Fang C Kim H Yanagisawa L Bennett W Sirven MA Alaniz RC. Gallotannins and *lactobacillus plantarum* WCFS1 mitigate high-fat diet-induced inflammation and induce biomarkers for thermogenesis in adipose tissue in gnotobiotic mice. Mol Nutr Food Res. (2019) 63:e1800937. doi: 10.1002/mnfr.201800937, PMID: 30908878

[8] Liu W-H Chuang H-L Huang Y-T Wu C-C Chou G-T Wang S. Alteration of behavior and monoamine levels attributable to *Lactobacillus plantarum* PS128 in germ-free mice. Behav Brain Res. (2016) 298:202–9. doi: 10.1016/j.bbr.2015.10.046, PMID: 26522841

[9] Kozakova H Schwarzer M Srutkova D Hudcovic T Cukrowska B. Colonisation of germ-free mice with probiotic lactobacilli mitigated allergic sensitisation in murine model of birch pollen allergy. Clin Transl Allergy. (2014) 4:26. doi: 10.1186/2045-7022-4-s2-p2625225607

[10] Fåk F Ahrné S Molin G Jeppsson B Weström B. Maternal consumption of *Lactobacillus plantarum* 299v affects gastrointestinal growth and function in the suckling rat. Br. J. Nutr. (2008) 100:332–8. doi: 10.1017/s0007114507883036 18179726

[11] OjiNjideka Hemphill N Pezley L Steffen A Elam G Kominiarek MA Odoms-Young A. Feasibility study of *Lactobacillus plantarum* 299v probiotic supplementation in an urban academic facility among diverse pregnant individuals. Nutrients. (2023) 15:875. doi: 10.3390/nu15040875, PMID: 36839232 PMC9966742

[12] Wang Y Zhang Z Liu B Zhang C Zhao J Li X. A study on the method and effect of the construction of a humanized mouse model of fecal microbiota transplantation. Front Microbiol. (2022) 13:1031758. doi: 10.3389/fmicb.2022.1031758, PMID: 36466673 PMC9709132

[13] Truong DT Franzosa EA Tickle TL Scholz M Weingart G Pasolli E. MetaPhlAn2 for enhanced metagenomic taxonomic profiling. Nat Methods. (2015) 12:902–3. doi: 10.1038/nmeth.3589, PMID: 26418763

[14] Franzosa EA McIver LJ Rahnavard G Thompson LR Schirmer M Weingart G. Species-level functional profiling of metagenomes and metatranscriptomes. Nat Methods. (2018) 15:962–8. doi: 10.1038/s41592-018-0176-y, PMID: 30377376 PMC6235447

[15] Li D Liu C-M Luo R Sadakane K Lam T-W. MEGAHIT: an ultra-fast single-node solution for large and complex metagenomics assembly via succinct de Bruijn graph. Bioinformatics. (2015) 31:1674–6. doi: 10.1093/bioinformatics/btv033, PMID: 25609793

[16] Hyatt D Chen G-L LoCascio PF Land ML Larimer FW Hauser LJ. Prodigal: prokaryotic gene recognition and translation initiation site identification. BMC Bioinformatics. (2010) 11:119. doi: 10.1186/1471-2105-11-119, PMID: 20211023 PMC2848648

[17] Kang DD Li F Kirton E Thomas A Egan R An H. MetaBAT 2: an adaptive binning algorithm for robust and efficient genome reconstruction from metagenome assemblies. PeerJ. (2019) 7:e7359. doi: 10.7717/peerj.7359, PMID: 31388474 PMC6662567

[18] Parks DH Imelfort M Skennerton CT Hugenholtz P Tyson GW. CheckM: assessing the quality of microbial genomes recovered from isolates, single cells, and metagenomes. Genome Res. (2015) 25:1043–55. doi: 10.1101/gr.186072.114, PMID: 25977477 PMC4484387

[19] Chaumeil P-A Mussig AJ Hugenholtz P Parks DH. GTDB-Tk: a toolkit to classify genomes with the genome taxonomy database. Bioinformatics. (2019) 36:1925–7. doi: 10.1093/bioinformatics/btz848, PMID: 31730192 PMC7703759

[20] Hsu Y-L Chen C-C Lin Y-T Wu W-K Chang L-C Lai C-H. Evaluation and optimization of sample handling methods for quantification of short-chain fatty acids in human fecal samples by GC–MS. J Proteome Res. (2019) 18:1948–57. doi: 10.1021/acs.jproteome.8b00536, PMID: 30895795

[21] Chen G-Y Zhong W Zhou Z Zhang Q. Simultaneous determination of tryptophan and its 31 catabolites in mouse tissues by polarity switching UHPLC-SRM-MS. Anal Chim Acta. (2018) 1037:200–10. doi: 10.1016/j.aca.2018.02.026, PMID: 30292294 PMC6224157

[22] Yang T Shu T Liu G Mei H Zhu X Huang X. Quantitative profiling of 19 bile acids in rat plasma, liver, bile and different intestinal section contents to investigate bile acid homeostasis and the application of temporal variation of endogenous bile acids. J Steroid Biochem Mol Biol. (2017) 172:69–78. doi: 10.1016/j.jsbmb.2017.05.015, PMID: 28583875

[23] Le TT Shafaei A Genoni A Christophersen C Devine A Lo J. Development and validation of a simple LC-MS/MS method for the simultaneous quantitative determination of trimethylamine-N-oxide and branched chain amino acids in human serum. Anal Bioanal Chem. (2019) 411:1019–28. doi: 10.1007/s00216-018-1522-8, PMID: 30552494

[24] Zelena E Dunn WB Broadhurst D Francis-McIntyre S Carroll KM Begley P. Development of a robust and repeatable UPLC-MS method for the long-term metabolomic study of human serum. Anal Chem. (2009) 81:1357–64. doi: 10.1021/ac8019366, PMID: 19170513

[25] Adusumilli R Mallick P. Data conversion with ProteoWizard msConvert. New York: Springer, pp. 339–368. (2017). 10.1007/978-1-4939-6747-6_23 28188540

[26] Domingo-Almenara X Siuzdak G. Metabolomics data processing using XCMS. Berlin: Springer US, pp. 11–24. (2020). 10.1007/978-1-0716-0239-3_2 31953810

[27] R Core Team. *R: A language and environment for statistical computing*. (2015). Available at: http://www.r-project.org/

[28] Liu C Cui Y Li X Yao M. Microeco: an R package for data mining in microbial community ecology. FEMS Microbiol Ecol. (2021) 97:1–19. doi: 10.1093/femsec/fiaa255 33332530

[29] Lu Y Zhou G Ewald J Pang Z Shiri T Xia J. MicrobiomeAnalyst 2.0: comprehensive statistical, functional and integrative analysis of microbiome data. Nucleic Acids Res. (2023) 51:W310–8. doi: 10.1093/nar/gkad407, PMID: 37166960 PMC10320150

[30] Pang Z Zhou G Ewald J Chang L Hacariz O Basu N. Using MetaboAnalyst 5.0 for LC–HRMS spectra processing, multi-omics integration and covariate adjustment of global metabolomics data. Nat Protoc. (2022) 17:1735–61. doi: 10.1038/s41596-022-00710-w, PMID: 35715522

[31] Cheng Y-C Liu J-R. Effect of *Lactobacillus rhamnosus* GG on energy metabolism, leptin resistance, and gut microbiota in mice with diet-induced obesity. Nutrients. (2020) 12:2557. doi: 10.3390/nu12092557, PMID: 32846917 PMC7551584

[32] Wang G Wang X Ma Y Cai S Yang L Fan Y. *Lactobacillus* reuteri improves the development and maturation of fecal microbiota in piglets through mother-to-infant microbe and metabolite vertical transmission. Microbiome. (2022) 10:211. doi: 10.1186/s40168-022-01336-6, PMID: 36461096 PMC9717520

[33] Wang M Wu H Lu L Jiang L Yu Q. *Lactobacillus reuteri* promotes intestinal development and regulates mucosal immune function in newborn piglets. Front Vet Sci. (2020) 7:42. doi: 10.3389/fvets.2020.00042, PMID: 32118065 PMC7018766

[34] Sang J Zhuang D Zhang T Wu Q Yu J Zhang Z. Convergent and divergent age patterning of gut microbiota diversity in humans and nonhuman Primates. Msystems. (2022) 7:e0151221–1. doi: 10.1128/msystems.01512-21, PMID: 35758593 PMC9426537

[35] Chen M Guo W-L Li Q-Y Xu J-X Cao Y-J Liu B. The protective mechanism of *Lactobacillus plantarum* FZU3013 against non-alcoholic fatty liver associated with hyperlipidemia in mice fed a high-fat diet. Food Funct. (2020) 11:3316–31. doi: 10.1039/C9FO03003D, PMID: 32226996

[36] Huang Y Wu Y Jia X Lin J Xiao L Liu D. *Lactiplantibacillus plantarum* DMDL 9010 alleviates dextran sodium sulfate (DSS)-induced colitis and behavioral disorders by facilitating microbiota-gut-brain axis balance. Food Funct. (2022) 13:411–24. doi: 10.1039/d1fo02938j, PMID: 34913458

[37] Wang L Zhao Z Zhao L Zhao Y Yang G Wang C. *Lactobacillus plantarum* DP189 reduces α-SYN aggravation in MPTP-induced Parkinson’s disease mice via regulating oxidative damage, inflammation, and gut microbiota disorder. J Agric Food Chem. (2022) 70:1163–73. doi: 10.1021/acs.jafc.1c07711 35067061

[38] Li M Wu X Guo Z Gao R Ni Z Cui H. *Lactiplantibacillus plantarum* enables blood urate control in mice through degradation of nucleosides in gastrointestinal tract. Microbiome. (2023) 11:153. doi: 10.1186/s40168-023-01605-y, PMID: 37468996 PMC10354915

[39] Heeney D Barouei J Hsieh Y Martinic A Mishchuk D Kieffer D. *Lactobacillus plantarum* improves metabolic outcomes and alters the colonic immune state in mice fed a high fat diet. FASEB J. (2016) 30:854. doi: 10.1096/fasebj.30.1_supplement.854.1

[40] Szczuko M Kikut J Maciejewska D Kulpa D Celewicz Z Ziętek M. The associations of SCFA with anthropometric parameters and carbohydrate metabolism in pregnant women. Int J Mol Sci. (2020) 21:9212. doi: 10.3390/ijms21239212 33287163 PMC7731050

[41] Wu Y Jha R Li A Liu H Zhang Z Zhang C. Probiotics (*Lactobacillus plantarum* HNU082) supplementation relieves ulcerative colitis by affecting intestinal barrier functions, immunity-related gene expression, gut microbiota, and metabolic pathways in mice. Microbiol Spectr. (2022) 10:e0165122. doi: 10.1128/spectrum.01651-22, PMID: 36321893 PMC9769980

[42] Yamasaki M Minesaki M Iwakiri A Miyamoto Y Ogawa K Nishiyama K. *Lactobacillus plantarum* 06CC2 reduces hepatic cholesterol levels and modulates bile acid deconjugation in Balb/c mice fed a high-cholesterol diet. Food Sci Nutr. (2020) 8:6164–73. doi: 10.1002/fsn3.1909, PMID: 33282267 PMC7684586

[43] Lee Y-S Lee D Park G-S Ko S-H Park J Lee Y-K. *Lactobacillus plantarum* HAC01 ameliorates type 2 diabetes in high-fat diet and streptozotocin-induced diabetic mice in association with modulating the gut microbiota. Food Funct. (2021) 12:6363–73. doi: 10.1039/d1fo00698c, PMID: 34105563

[44] Liang C Zhou X-H Gong P-M Niu H-Y Lyu L-Z Wu Y-F. Lactiplantibacillus plantarum H-87 prevents high-fat diet-induced obesity by regulating bile acid metabolism in C57BL/6J mice. Food Funct. (2021) 12:4315–24. doi: 10.1039/D1FO00260K, PMID: 34031676

[45] Wang W Liu F Xu C Liu Z Ma J Gu L. *Lactobacillus plantarum* 69-2 combined with galacto-oligosaccharides alleviates d-galactose-induced aging by regulating the AMPK/SIRT1 signaling pathway and gut microbiota in mice. J Agric Food Chem. (2021) 69:2745–57. doi: 10.1021/acs.jafc.0c06730, PMID: 33565862

[46] Graeff FG Guimarães FS De Andrade TGCS Deakin JFW. Role of 5-HT in stress, anxiety, and depression. Pharmacol Biochem Behav. (1996) 54:129–41. doi: 10.1016/0091-3057(95)02135-3 8728550

[47] Pawluski JL Li M Lonstein JS. Serotonin and motherhood: from molecules to mood. Front Neuroendocrinol. (2019) 53:100742. doi: 10.1016/j.yfrne.2019.03.001, PMID: 30878665 PMC6541513

[48] Shi J Du P Xie Q Wang N Li H Smith EE. Protective effects of tryptophan-catabolizing *Lactobacillus plantarum* KLDS 1.0386 against dextran sodium sulfate-induced colitis in mice. Food Funct. (2020) 11:10736–47. doi: 10.1039/d0fo02622k, PMID: 33231244

[49] Wang A Guan C Wang T Mu G Tuo Y. Indole-3-lactic acid, a tryptophan metabolite of *Lactiplantibacillus plantarum* DPUL-S164, improved intestinal barrier damage by activating AhR and nrf2 signaling pathways. J Agr Food Chem. (2023) 71:18792–801. doi: 10.1021/acs.jafc.3c06183, PMID: 37996788

[50] Gayathri D Rashmi BS. Mechanism of development of depression and probiotics as adjuvant therapy for its prevention and management. Ment Health Prevent. (2017) 5:40–51. doi: 10.1016/j.mhp.2017.01.003

[51] Ramireddy L Tsen H-Y Chiang Y-C Hung C-Y Wu S-R Young S-L. Molecular identification and selection of probiotic strains able to reduce the serum TMAO level in mice challenged with choline. Food Secur. (2021) 10:2931. doi: 10.3390/foods10122931, PMID: 34945482 PMC8700464

[52] Ma F Sun M Song Y Wang A Jiang S Qian F. *Lactiplantibacillus plantarum*-12 alleviates inflammation and Colon Cancer symptoms in AOM/DSS-treated mice through modulating the intestinal microbiome and metabolome. Nutrients. (2022) 14:1916. doi: 10.3390/nu14091916, PMID: 35565884 PMC9100115

[53] Wu Y Li A Liu H Zhang Z Zhang C Ma C. *Lactobacillus plantarum* HNU082 alleviates dextran sulfate sodium-induced ulcerative colitis in mice through regulating gut microbiome. Food Funct. (2022) 13:10171–85. doi: 10.1039/d2fo02303b, PMID: 36111438

[54] Li C Cao J Nie S-P Zhu K-X Xiong T Xie M-Y. Serum metabolomics analysis for biomarker of *Lactobacillus plantarum* NCU116 on hyperlipidaemic rat model feed by high fat diet. J Funct Foods. (2018) 42:171–6. doi: 10.1016/j.jff.2017.12.036

[55] Sakai T Taki T Nakamoto A Shuto E Tsutsumi R Toshimitsu T. *Lactobacillus plantarum* OLL2712 regulates glucose metabolism in C57BL/6 mice fed a high-fat diet. J Nutr Sci Vitaminol. (2013) 59:144–7. doi: 10.3177/jnsv.59.144, PMID: 23727645

[56] Prescott SL Wickens K Westcott L Jung W Currie H Black PN. Supplementation with *Lactobacillus rhamnosus* or *Bifidobacterium lactis* probiotics in pregnancy increases cord blood interferon-γ and breast milk transforming growth factor-β and immunoglobin a detection. Clin Exp Allergy. (2008) 38:1606–14. doi: 10.1111/j.1365-2222.2008.03061.x, PMID: 18631345

[57] Navarro-Tapia E Sebastiani G Sailer S Almeida Toledano L Serra-Delgado M García-Algar Ó. Probiotic supplementation during the perinatal and infant period: effects on gut dysbiosis and disease. Nutrients. (2020) 12:2243. doi: 10.3390/nu12082243, PMID: 32727119 PMC7468726

[58] Obuchowska A Gorczyca K Standyło A Obuchowska K Kimber-Trojnar Ż Wierzchowska-Opoka M. Effects of probiotic supplementation during pregnancy on the future maternal risk of metabolic syndrome. Int J Mol Sci. (2022) 23:8253. doi: 10.3390/ijms23158253, PMID: 35897822 PMC9330652

[59] Treven P Mrak V Bogovič Matijašić B Horvat S Rogelj I. Administration of probiotics *Lactobacillus rhamnosus* GG and *Lactobacillus gasseri* K7 during pregnancy and lactation changes mouse mesenteric lymph nodes and mammary gland microbiota. J Dairy Sci. (2015) 98:2114–28. doi: 10.3168/jds.2014-8519, PMID: 25622869

[60] Yu Y Lu J Oliphant K Gupta N Claud K Lu L. Maternal administration of probiotics promotes gut development in mouse offsprings. PLoS One. (2020) 15:e0237182. doi: 10.1371/journal.pone.0237182, PMID: 32764797 PMC7413491

[61] Azagra-Boronat I Tres A Massot-Cladera M Franch À Castell M Guardiola F. *Lactobacillus fermentum* CECT5716 supplementation in rats during pregnancy and lactation impacts maternal and offspring lipid profile, immune system and microbiota. Cells. (2020) 9:575. doi: 10.3390/cells9030575, PMID: 32121244 PMC7140451

