# Supplementary material for: Lactiplantibacillusplantarum HM-P2 influences gestational gut microbiome and microbial metabolism
Source: Front Nutr. 2024 Dec 20;11:1489359. doi: 10.3389/fnut.2024.1489359 (PMC11695228; doi:10.3389/fnut.2024.1489359)
Supplement: Supplementary file 1 [file Supplementary_file_1.docx]

**Supplement File 1. Summary of *Lactiplantibacillus plantarum* HM-P2’s probiotic properties.**

As mentioned in our published works and granted patents: Briefly, HM-P2 demonstrates high bile salt tolerance, with a survival rate of ≥118.2% after 1 hour and ≥172.4% after 3 hours in 0.3% bile salts, enabling it to withstand the high bile salt environment of the human small intestine. It also shows good acid tolerance, with survival rates of 166% and 139% after 1 hour and 3 hours, respectively, in an environment with pH 3.0. Additionally, HM-P2 exhibits strong antibacterial activity against pathogenic *Escherichia coli* and/or *Staphylococcus aureus*, with inhibition zone diameters of 26.21 ± 0.52 mm for *E. coli* and 24.30 ± 0.52 mm for *S. aureus*. The adhesion rate of HM-P2 is 120%. The strain is sensitive to 13 antibiotics, including cephalosporins, ampicillin, carbapenems, macrolides, and clindamycin, but resistant to aminoglycosides, glycopeptides, and fluoroquinolones. No virulence factors were detected in the virulence factor database (VFDB).

Our published works and granted patents related with HM-P2:

1. “Breast milk source Lactobacillus plantarum HM-P2 and application thereof, CN115491329A”, (English version), https://patents.google.com/patent/CN115491329A/en?oq=CN115491329A.
2. PCT patent “Breast milk-derived lactobacillus plantarum hm-p2 and use thereof, WO2024060768”. https://patentscope2.wipo.int/search/en/detail.jsf?docId=WO2024060768&_cid=P20-LV8O1F-82651-1.
3. YIN C M, LI Z, JIANG T M, et al. Screening and identification of ability lactic acid bacteria from human breast milk and preliminary determination of its ability to hypotensive[J]. Food Science and Technology,2019,44(8):18−22. (In Chinese). <https://qikan.cqvip.com/Qikan/Article/Detail?id=7002912528>.
4. “Breast milk source Lactobacillus plantarum and application thereof”, CN111778180B, (English version), <https://patents.google.com/patent/CN111778180B/en>.”
