# Supplementary material for: Lactiplantibacillusplantarum HM-P2 influences gestational gut microbiome and microbial metabolism
Source: Front Nutr. 2024 Dec 20;11:1489359. doi: 10.3389/fnut.2024.1489359 (PMC11695228; doi:10.3389/fnut.2024.1489359)
Supplement: Supplementary file 2 [file Supplementary_file_2.docx]

**Supplement File 2. Summary of *Lactiplantibacillus plantarum* HM-P2’s dosage selection.**

The final dose selection was determined based on our patent data and existing literature. Briefly, The dose selection was based on a preliminary evaluation using three dosage groups in mice: high (10¹⁰ CFU/mL), medium (10⁹ CFU/mL), and low (10⁸ CFU/mL), with the control group receiving 0.85% saline. Results showed no organ abnormalities, with normal color, soft texture, and absence of tumors. There were no significant differences (P > 0.05) in organ indices (heart, liver, spleen, lung), blood parameters, or serum biochemical markers between the experimental and control groups. Additionally, no bacterial translocation was observed, as no colonies grew on MRS medium plated with organ samples.

The dosages of *Lactobacillus plantarum* in mice studies vary widely depending on the strain and the specific health outcomes being targeted. Common dosages range from 10^7 to 10^10 CFU/mL, with higher dosages often used to ensure safety and persistence in the gastrointestinal tract. For example, *Lactobacillus plantarum* TWK10 was administered at 2.05 × 10^8 CFU/kg/day and 1.03 × 10^9 CFU/kg/day to improve exercise performance and muscle mass [2]. *Lactiplantibacillus plantarum* 2-33 was administered at 1.0 × 10^7 CFU/mL, 1.0 × 10^8 CFU/mL, and 1.0 × 10^9 CFU/mL in antibiotic-associated diarrhea (AAD) mice [3].

Based on these results and literature data, the medium dose (10⁹ CFU/mL) was selected for the FMT experiment.

References:

1. “Breast milk source Lactobacillus plantarum HM-P2 and application thereof, CN115491329A”, (English version), <https://patents.google.com/patent/CN115491329A/en?oq=CN115491329A>.
2. Chen YM, Wei L, Chiu YS, Hsu YJ, Tsai TY, Wang MF, Huang CC. Lactobacillus plantarum TWK10 Supplementation Improves Exercise Performance and Increases Muscle Mass in Mice. Nutrients. 2016 Apr 7;8(4):205. doi: 10.3390/nu8040205. PMID: 27070637; PMCID: PMC4848674.
3. Bao W, He Y, Yu J, Liu M, Yang X, Ta N, Zhang E and Liang C (2022) Regulatory Effect of *Lactiplantibacillus plantarum* 2-33 on Intestinal Microbiota of Mice With Antibiotic-Associated Diarrhea. *Front. Nutr.* 9:921875. doi: 10.3389/fnut.2022.921875
