# Supplementary material for: Lactiplantibacillusplantarum HM-P2 influences gestational gut microbiome and microbial metabolism
Source: Front Nutr. 2024 Dec 20;11:1489359. doi: 10.3389/fnut.2024.1489359 (PMC11695228; doi:10.3389/fnut.2024.1489359)
Supplement: Supplementary file 3 [file Table_1.docx]

**Table S1. Enrichment analysis of differentially expressed metagenomic genes (KOs) , related to Results.**

| Pathway | Total | Expected | Hits | Pval | FDR |
| --- | --- | --- | --- | --- | --- |
| Starch and sucrose metabolism | 73 | 0.545 | 5 | 0.000159 | 0.0239 |
| Fatty acid biosynthesis | 28 | 0.209 | 2 | 0.0179 | 1 |
| Propanoate metabolism | 82 | 0.612 | 3 | 0.022 | 1 |
| Biosynthesis of various plant secondary metabolites | 7 | 0.0522 | 1 | 0.0511 | 1 |
| Phenylalanine, tyrosine and tryptophan biosynthesis | 58 | 0.433 | 2 | 0.0684 | 1 |
| Nicotinate and nicotinamide metabolism | 66 | 0.492 | 2 | 0.0857 | 1 |
| Cyanoamino acid metabolism | 16 | 0.119 | 1 | 0.113 | 1 |
| Terpenoid backbone biosynthesis | 23 | 0.172 | 1 | 0.159 | 1 |
| Cysteine and methionine metabolism | 97 | 0.724 | 2 | 0.162 | 1 |
| Carbon fixation in photosynthetic organisms | 26 | 0.194 | 1 | 0.178 | 1 |
