# Supplementary material for: Lactiplantibacillusplantarum HM-P2 influences gestational gut microbiome and microbial metabolism
Source: Front Nutr. 2024 Dec 20;11:1489359. doi: 10.3389/fnut.2024.1489359 (PMC11695228; doi:10.3389/fnut.2024.1489359)
Supplement: Supplementary file 4 [file Table_2.docx]

**Table S2. Difference analysis of tissue fresh weight, related to Results.**

| fresh weight, g | Group | N | Mean | Median | SD | p ^a^ | Effect Size ^b^ |
| --- | --- | --- | --- | --- | --- | --- | --- |
| liver, total | control | 4 | 1.6104 | 1.6448 | 0.19198 | 0.106 | 0.6111 |
|  | L. plantarum HM-P2 | 6 | 1.4374 | 1.4476 | 0.11351 |  |  |
| left lobe of liver | control | 4 | 0.5102 | 0.4925 | 0.08337 | 0.414 | 0.3333 |
|  | L. plantarum HM-P2 | 6 | 0.4632 | 0.46 | 0.04322 |  |  |
| spleen | control | 4 | 0.0626 | 0.0609 | 0.00738 | 0.94 | 0.0556 |
|  | L. plantarum HM-P2 | 6 | 0.0632 | 0.0623 | 0.00782 |  |  |
| left kidney | control | 4 | 0.1292 | 0.1276 | 0.00519 | 0.414 | 0.3333 |
|  | L. plantarum HM-P2 | 6 | 0.133 | 0.1381 | 0.01092 |  |  |
| right kidney | control | 4 | 0.1372 | 0.137 | 0.00738 | 0.414 | 0.3333 |
|  | L. plantarum HM-P2 | 6 | 0.1418 | 0.1456 | 0.01365 |  |  |

^a^, Mann-Whitney U. ^b^,Rank biserial correlation
