# Supplementary material for: Lactiplantibacillusplantarum HM-P2 influences gestational gut microbiome and microbial metabolism
Source: Front Nutr. 2024 Dec 20;11:1489359. doi: 10.3389/fnut.2024.1489359 (PMC11695228; doi:10.3389/fnut.2024.1489359)
Supplement: Supplementary file 5 [file Table_3.docx]

**Table S3. Difference analysis of cytokine and immunoglobulin levels, related to Results.**

|  | Group | N | Mean | Median | SD | p ^a^ | Effect Size ^b^ |
| --- | --- | --- | --- | --- | --- | --- | --- |
| TGFbeta1 | Control | 4 | 797.01 | 774.683 | 186.616 | 0.257 | 0.5 |
|  | L. plantarum HM-P2 | 6 | 924.517 | 839.749 | 221.624 |  |  |
| IFNgamma | Control | 4 | 414.312 | 341.064 | 292.65 | 0.476 | 0.3333 |
|  | L. plantarum HM-P2 | 6 | 496.9 | 562.039 | 195.73 |  |  |
| TNFalpha | Control | 4 | 386.401 | 301.952 | 309.769 | 0.762 | 0.1667 |
|  | L. plantarum HM-P2 | 6 | 404.041 | 367.128 | 167.648 |  |  |
| IL10 | Control | 4 | 156.166 | 158.069 | 65.708 | 0.669 | 0.2083 |
|  | L. plantarum HM-P2 | 6 | 193.103 | 207.026 | 59.259 |  |  |
| IL8 | Control | 4 | 193.19 | 155.877 | 166.604 | 0.914 | 0.0833 |
|  | L. plantarum HM-P2 | 6 | 184.971 | 172.933 | 82.533 |  |  |
| IL6 | Control | 4 | 22.28 | 17.911 | 17.686 | 0.914 | 0.0833 |
|  | L. plantarum HM-P2 | 6 | 21.006 | 19.046 | 9.684 |  |  |
| IL4 | Control | 4 | 53.566 | 66.657 | 29.585 | 0.831 | 0.125 |
|  | L. plantarum HM-P2 | 6 | 60.701 | 59.135 | 22.717 |  |  |
| TGFalpha | Control | 4 | 332.625 | 251.194 | 212.205 | 1 | 0 |
|  | L. plantarum HM-P2 | 6 | 329.489 | 285.494 | 191.435 |  |  |
| IL22 | Control | 4 | 14.927 | 9.354 | 17.692 | 0.914 | 0.0833 |
|  | L. plantarum HM-P2 | 6 | 12.417 | 11.42 | 4.448 |  |  |
| IL17 | Control | 4 | 363.525 | 364.73 | 286.655 | 0.762 | 0.1667 |
|  | L. plantarum HM-P2 | 6 | 442.326 | 385.762 | 190.883 |  |  |
| IL1beta | Control | 4 | 149.333 | 159.417 | 83.655 | 0.762 | 0.1667 |
|  | L. plantarum HM-P2 | 6 | 166.121 | 171.928 | 82.202 |  |  |
| IL1alpha | Control | 4 | 124.933 | 125.046 | 105.374 | 0.914 | 0.0833 |
|  | L. plantarum HM-P2 | 6 | 102.377 | 75.455 | 60.266 |  |  |
| IgM | Control | 4 | 0.638 | 0.651 | 0.489 | 0.914 | 0.0833 |
|  | L. plantarum HM-P2 | 6 | 0.504 | 0.477 | 0.232 |  |  |
| IgG | Control | 4 | 34.563 | 21.468 | 37.596 | 0.914 | 0.0833 |
|  | L. plantarum HM-P2 | 6 | 16.841 | 14.735 | 6.945 |  |  |

^a^, Mann-Whitney U. ^b^,Rank biserial correlation
