# Supplementary figures and images for: Lactiplantibacillusplantarum HM-P2 influences gestational gut microbiome and microbial metabolism
Source: Front Nutr. 2024 Dec 20;11:1489359. doi: 10.3389/fnut.2024.1489359 (PMC11695228; doi:10.3389/fnut.2024.1489359)

Comparison of offspring number in the control and HM-P2 groups.

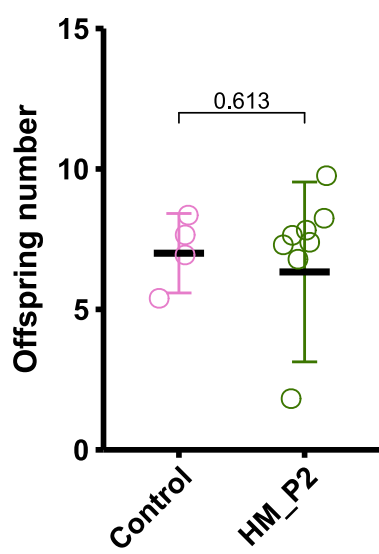

Supplement: Supplementary file 6 [file Image_1.pdf]

Heatmap of SCFA contents in fecal from 0d to 18d of gestation.

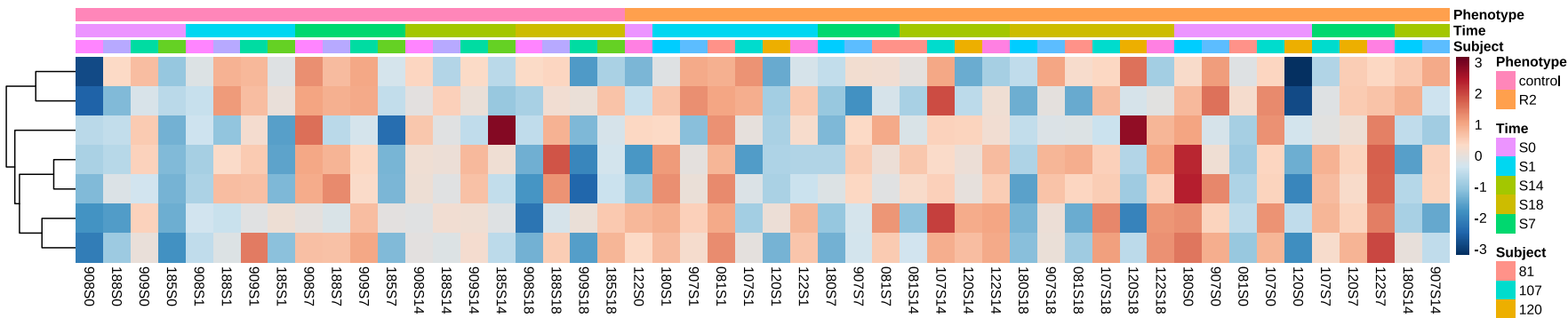

<sup>a</sup>, R2, *L. plantarum* HM-P2

Supplement: Supplementary file 7 [file Image_2.pdf]

Heatmap of tryptophan contents in cecum at 18d of gestation.

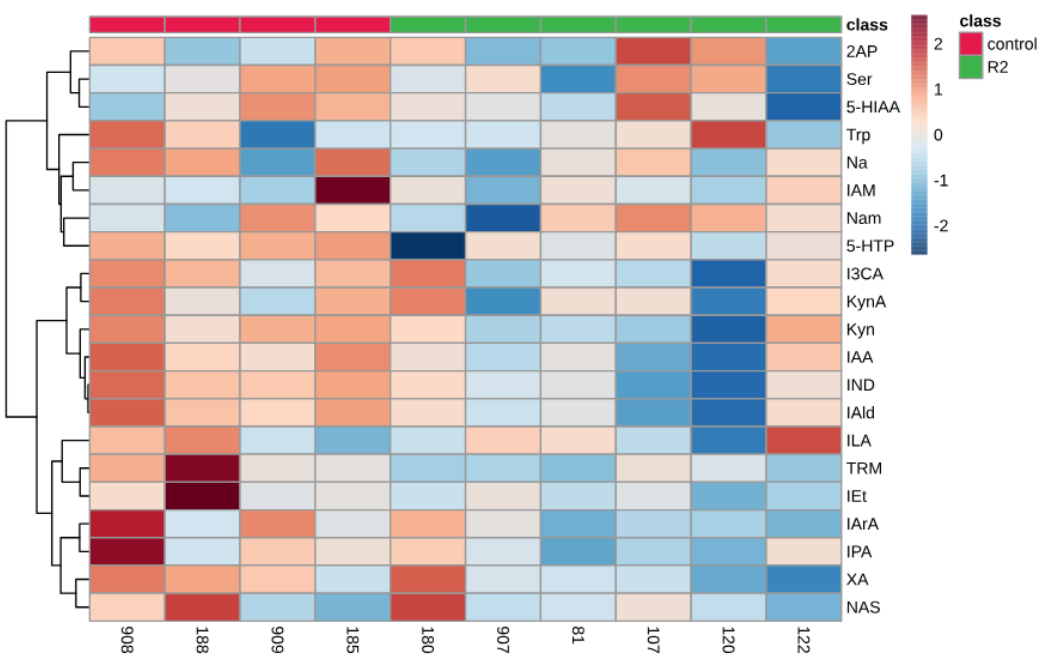

<sup>a</sup>, R2, *L. plantarum* HM-P2

Supplement: Supplementary file 8 [file Image_3.pdf]

1    **Heatmap of TMAO contents in serum at 18d of gestation.**

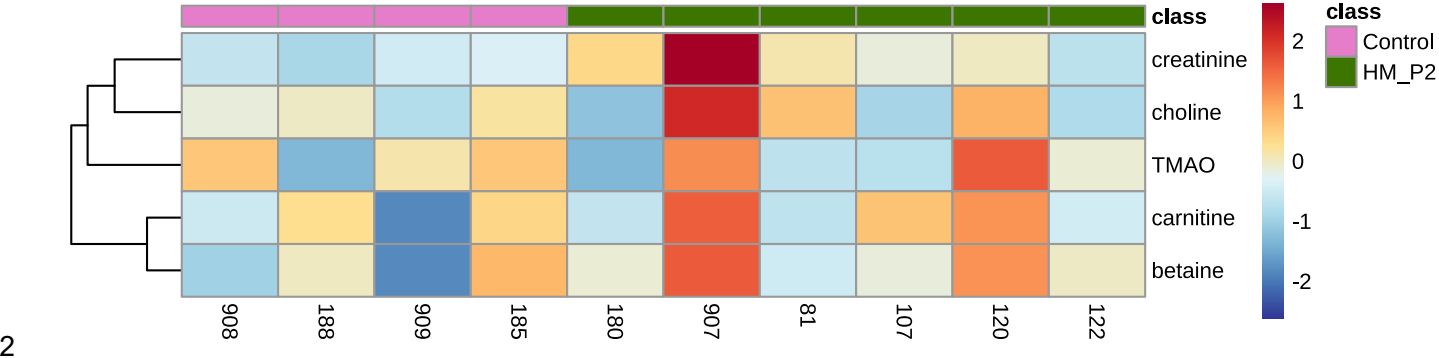

Supplement: Supplementary file 9 [file Image_4.pdf]
